# Mesoscale Assembly of Bisteroidal Esters from Terephthalic Acid

**DOI:** 10.3390/molecules25051213

**Published:** 2020-03-08

**Authors:** Gabriel Guerrero-Luna, María Guadalupe Hernández-Linares, Sylvain Bernès, Alan Carrasco-Carballo, Diana Montalvo-Guerrero, María A. Fernández-Herrera, Jesús Sandoval-Ramírez

**Affiliations:** 1 Facultad de Ciencias Químicas, Benemérita Universidad Autónoma de Puebla, 72570 Puebla, Pue., Mexico; gabrielgluna@hotmail.com (G.G.-L.); alcdack@gmail.com (A.C.-C.); jesus.sandoval@correo.buap.mx (J.S.-R.); 2 Centro de Química, Instituto de Ciencias, Benemérita Universidad Autónoma de Puebla, 72570 Puebla, Pue., Mexico; 3 Laboratorio de Investigación, Herbario y Jardín Botánico Universitario, Benemérita Universidad Autónoma de Puebla, 72570 Puebla, Pue., Mexico; 4 Instituto de Física, Benemérita Universidad Autónoma de Puebla, 72570 Puebla, Pue., Mexico; sylvain_bernes@hotmail.com; 5 Departamento de Física Aplicada, Centro de Investigación y de Estudios Avanzados–Unidad Mérida, km 6 Antigua Carretera a Progreso, Cordemex, 97310 Mérida, Yuc., Mexico; diana.montalvo@cinvestav.mx (D.M.-G.); marietafernandezh@gmail.com (M.A.F.-H.)

**Keywords:** sapogenins, bisteroidal terephthalic esters, spacer groups, self-assembly behaviour

## Abstract

A new series of bisteroidal esters was synthesized using a spacer group, sterols and sapogenins as substrates. Steroidal dimers were prepared in high yields employing diesters of terephthalic acid as linkages at the 3β, 3′β steroidal positions. In all attempts to crystallize bisteroids, it was observed that the compounds tended to self-organize in solution, which was detected when employing various solvent systems. The non-covalent interactions (van der Waals) of the steroidal moieties of this series of symmetrical bisteroids, the polarity of the solvents systems, and the different solubilities of the bisteroid aggregates, indeed induce the molecules to self-assemble into supramolecular structures with well-defined organization. Our results show that the self-assembled structures for the bisteroidal derivatives depend on the solvent system used: with hexane/EtOAc, membrane-shaped structures were obtained, while pure EtOAc afforded strand-shaped arrangements. In the CHCl_3_/CH_3_OH system, thin strands were formed, since van der Waals interactions are lowered in this system, as a consequence of the increased solubility of the bisteroids in CHCl_3_. Based on the characterization by SEM and XRD, we show evidence that the phenomenon of self-assembly of bisteroids occurs presenting different morphologies depending on the solvent used. The new steroidal dimer derivatives were characterized by NMR, TGA, DSC, SEM, and XRD. Finally, the molecular structure of one bisteroid was confirmed by single-crystal X-ray analysis.

## 1. Introduction

In the chemistry of steroids, there is a constant interest in performing structural modifications on steroids to optimize their biological and pharmacological activities or to search new properties. Steroid dimers are a particular group that are well known for their rigid, predictable, and inherently asymmetric architecture [1].

**Figure 1:**
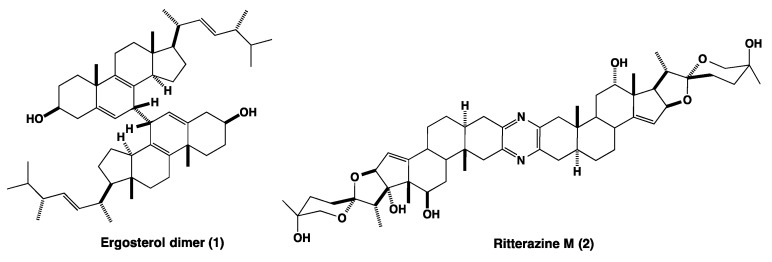
Photochemically formed dimer from ergosterol (**1**) and naturally occurring dimer Ritterazine M (**2**).

Steroid dimers belong to an essential group of pharmacologically active compounds that are predominantly biosynthesized by marine organisms [2,3]. The synthesis of steroid dimers was first identified throughout photochemical studies through the investigation of the effect of sensitized light on the activation of ergosterol, whose product was the ergosterol dimer (**1**, Figure 1) [4]. Bisteroidal conjugates caught great attention in the last years due to their noteworthy applications in biology, medicine, and supramolecular chemistry [5]. Examples of naturally occurring bisteroids are ritterazines and cephalostatins [6,7], which are marine natural products containing either two spirostanic or two spirodifuranic units connected by a pyrazine ring (**2**, Figure 1).

On the other hand, the making of functional materials and supramolecular systems employing naturally occurring molecules such as steroids has been gaining attention [8,9]. More recently, a growing interest in the design and synthesis of novel nano-objects with well-defined shapes has emerged [10]. However, despite extensive studies, it is still challenging to prepare nanoscale-assemblies with responsive behaviours employing this kind of substrate. Self-assembly strategies could provide innovative capabilities to biomaterials used in modern medicine and biotechnology. Recently, there has been an increased interest in the construction and design of new nano-architectural objects with well-defined shapes (nanoribbons [11], nanofibers [12], nanobelts [13], tubes [14], and rods [15]), in well-ordered and predictable sizes. Generally, a straight and efficient method for building these well-organized nanostructures is the control of supramolecular self-assembly by slightly altering environmental conditions, including solvent composition [16,17], temperature [18], pH changes [19,20], metal coordination [21], and light irradiation [22,23]. Consequent to their distinctive shapes, these nanostructures have shown potential applications, such as nanomaterials, supports for selective-catalysis, or templates to produce one-dimensional nanostructures [24]. Several steroid dimers have been synthesized [25,26], and among them, acyclic dimers involving connections through A, B, C, or D rings directly or through spacers, form the major group of such molecules; these dimers are also referred as ‘linear dimers’ [1]. The synthesis of dimeric steroids involving the A rings from the steroid monomers, connected through spacer groups, can be accomplished by linking through the most convenient position, C-3 [27]. The self-assembly of molecular entities with significant molecular weight with amphiphilic properties is a useful strategy for the formation of well-controlled materials [28,29,30]. Yang et al. reported that supramolecular structures formed by polyoxometalate–steroid conjugates in a self-assembly process can be greatly influenced by the molecular structures itself and solution components. In that case, engineering was performed by selecting the steroids (classically cholesterol, dehydrocholic, and cholic acid) and an organically modified Anderson-type POM cluster as building blocks to create steroid–POM–steroid hybrids [31]. Nakanishi et al. successfully prepared hierarchic nanoarchitectures through self-assembly of fullerene derivatives with different numbers of long hydrocarbon chains and semiperfluoro-alkyl tails [32]. Three-dimensional microparticles with nanoflakes at the external surfaces or microparticles with many plate-like units were obtained by the self-assembly of hydrocarbon-branched fullerene derivatives. On the other hand, the fullerene derivative with semiperfluoro-alkyl chains affords surfaces with water-repellency properties from its 1,1-diethoxyethane solution. These assembled structures may have applications on non-wetting, low adhesion and self-cleaning surfaces [33,34].

In the context of new synthetic approaches towards bisteroids, the primary objective of our research was to obtain new steroidal dimers from sapogenins, with the aim to investigate their characteristics and properties as biological activity. Our synthetic strategy involved the incorporation of a spacer group that would give new features to the steroid and that would make it stable and highly rigid for its application as a new material. Once we had obtained the different bisteroidal esters, we noticed that their behaviour in solution was not the typical one observed for a sapogeninic steroid. Possibly due to the amphiphilic nature of the steroidal moiety, the presence of the terephthalate group, and the high molecular weight of the bisteroid compared to the raw material, we discovered that they exhibit a self-assembly behaviour caused by the solvent. This unexpected phenomenon was exciting, and we began to study these compounds within the framework of materials, characterizing the assemblies and documenting the micro and macroscopic characteristics. Herein, we report the synthesis and characterization of seven steroidal dimers forming diesters linkages of the terephthalic acid at the 3β, 3′β steroidal positions in high yields. The chemical and physical characterization has been carried out through IR, NMR, thermogravimetric analysis (TGA), differential scanning calorimetry (DSC), scanning electron microscopy (SEM), powder X-ray diffraction (p-XRD) and single-crystal X-ray diffraction (XRD).

## 2. Results

### Synthesis and Purification of Bisteroidal Esters

**Scheme 1:**
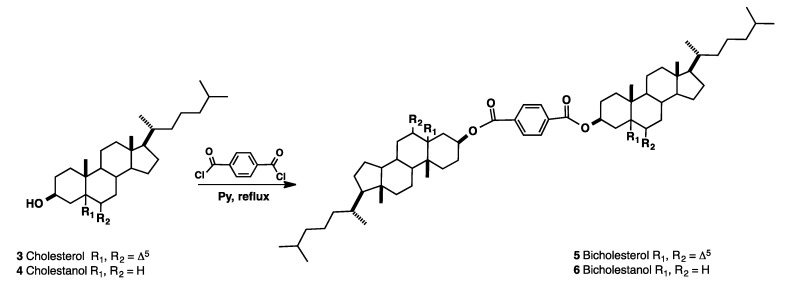
Dimeric sterol synthesis.

The synthesis of dimeric steroids was carried out by the construction of a terephthalate dimer containing a cholestane side chain or a spiroketal side chain in each repeat unit, that also, may allow us to change the functionality by stereoselectively opening the side chain. Bisteroids were obtained by the reaction of terephthaloyl chloride, acting as the spacer group, with the 3β-hydroxyl group of the steroid moiety, generating a tail-to-tail derivative, as shown in Scheme 1.

For the synthesis, different bases and solvents were tested at different temperatures (Table 1). Cholesterol and cholestanol moieties were selected as molecular targets because of the thermodynamic affinity of cholesterol for cell membranes and its ability to change its properties, for example by improving their mechanical durability and reducing passive permeability [35,36,37,38,39,40]; another advantage for cholestanes is their rigidity, a useful characteristic in nanoarchitecture.

The short-branched hydrocarbon tail confers to cholesterol a high hydrophobic structure, while the polar 3β-hydroxyl group provides a slight amphiphilic nature. The fused cyclohexane rings assume a puckered and more stable chair conformation, making cholesterol a planar and rigid structure, ideal for molecular architecture.

**Scheme 2:**
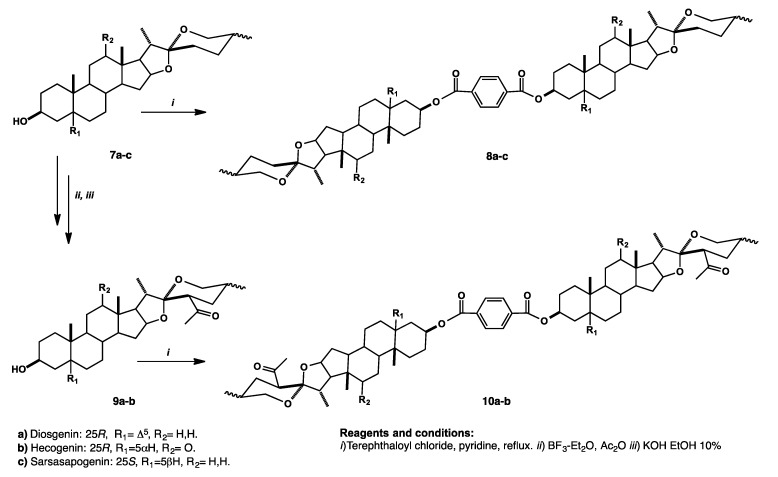
Synthetic route of bispirostanic derivatives **8a–c** and **10a–b**.

Next, dimeric steroids from diosgenin (**7a**), hecogenin (**7b**) and sarsasapogenin (**7c**) were also synthesized under the same conditions described in Table 1 entry **e**. Previously, we reported the transformation of (25*R*) and (25*S*)-sapogenins diosgenin (**a**), hecogenin (**b**) and sarsasapogenin (**c**) into the 23-acetyl-22, 26-epoxycholest-22-ene and (*E*)-20, 23-diacetylfurostenes, by means of Ac_2_O/Lewis acids at room temperature, which were then transformed into the 23-acetyl derivatives **9a–b** in good yields [41]. Taking in account the reactivity of rings E and F of sapogenins, the conditions of reaction were optimized, considering the lability of spiroketalic side chain in acid media, to obtain spirostanic dimers as diesteres of terephthalic acid (Scheme 2).

Most steroid dimers are nonpolar and can be separated by normal-phase column chromatography employing silica gel as the stationary phase and solvent mixtures based on *n*-hexane-EtOAc and CHCl_3_-MeOH, as eluent [42]. Alumina or celite as the stationary phase have also been used for the separation of other steroid dimers; in our work, the first attempt for purification of the crude was by flash chromatography. However, in some purifications, the crude of reaction supported on silica gel formed a self-organized “membrane” presumably by stimuli of the eluent (*n*-hexane-EtOAc), impeding the appropriate flow of solvent, and inducing the formation of strand-shaped aggregates of the compounds. In our subsequent work, for the initial separation of steroid dimers, solvent partitioning methods were employed.

## 3. Discussion

### 3.1. Structural Characterization of Bisteroidal Esters

#### 3.1.1. NMR Elucidation

**Figure 2:**
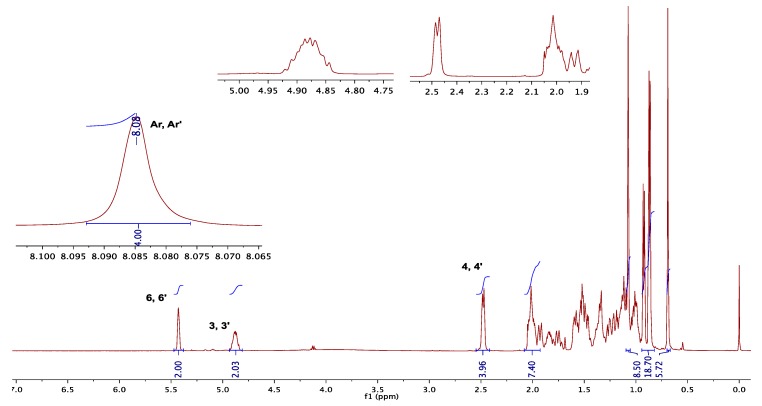
^1^H-NMR spectrum at 500 MHz of bicholesterol ester (**5**) in CDCl_3_.

The objective of using sterols of the 5α and 5β series as starting materials was to compare the difference in the NMR displacements in the steroidal moieties and the influence of the terephthalate group. The characterization by NMR of bicholesterol ester **5** is shown in Figure 2. Selected NMR signals for **5** and **6** are given in Table 2. The results obtained with cholesterol were useful to other Δ^5^ steroids as diosgenin.

**Figure 3:**
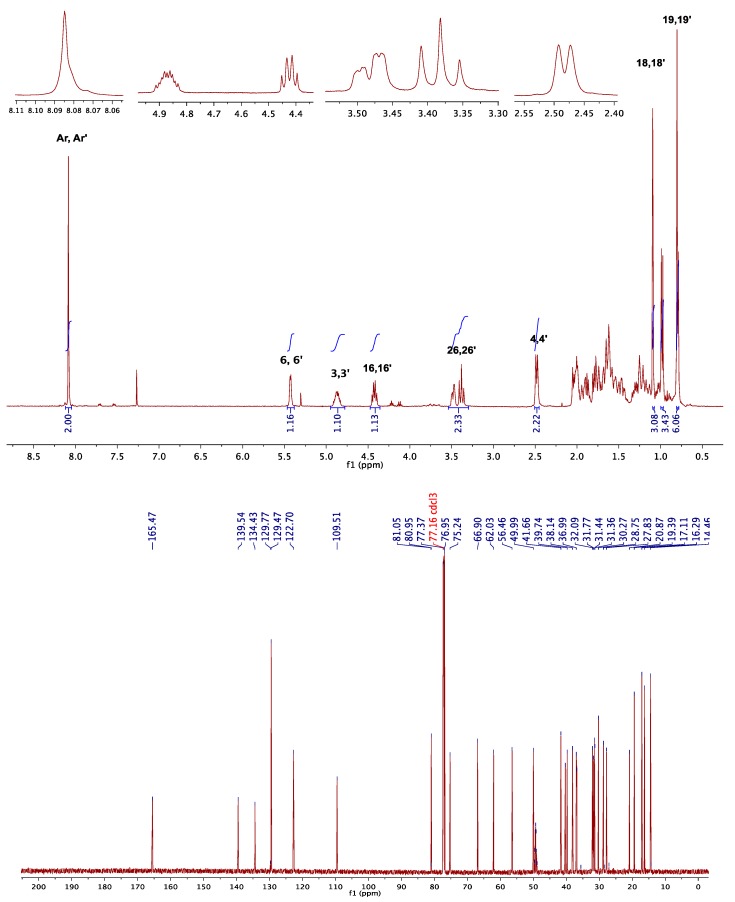
^1^H-NMR spectrum at 500 MHz in CDCl_3_ of **8a** with expansion in 8 ppm (top). ^13^C-NMR spectrum at 125 MHz in CDCl_3_ of **8a** (bottom).

By comparing the ^1^H- and ^13^C-NMR spectra for steroidal dimers **8a–c** and **10a–b** with the ones of the steroidal starting materials (sapogenins **7a–c** and **9a**–**b**), it was found that they are almost identical to those of the corresponding starting materials, except for the presence of signals attributable to the symmetrically 1,4-disubstituted-terephthalate bridge. Besides, when calculating the integration ratio of the singlet signal around 8 ppm belonging to the spacer group, this signal integrates for 4 H, showing that the aromatic ring is symmetrically disubstituted, as shown for **8a** (Figure 3).

**Figure 4:**
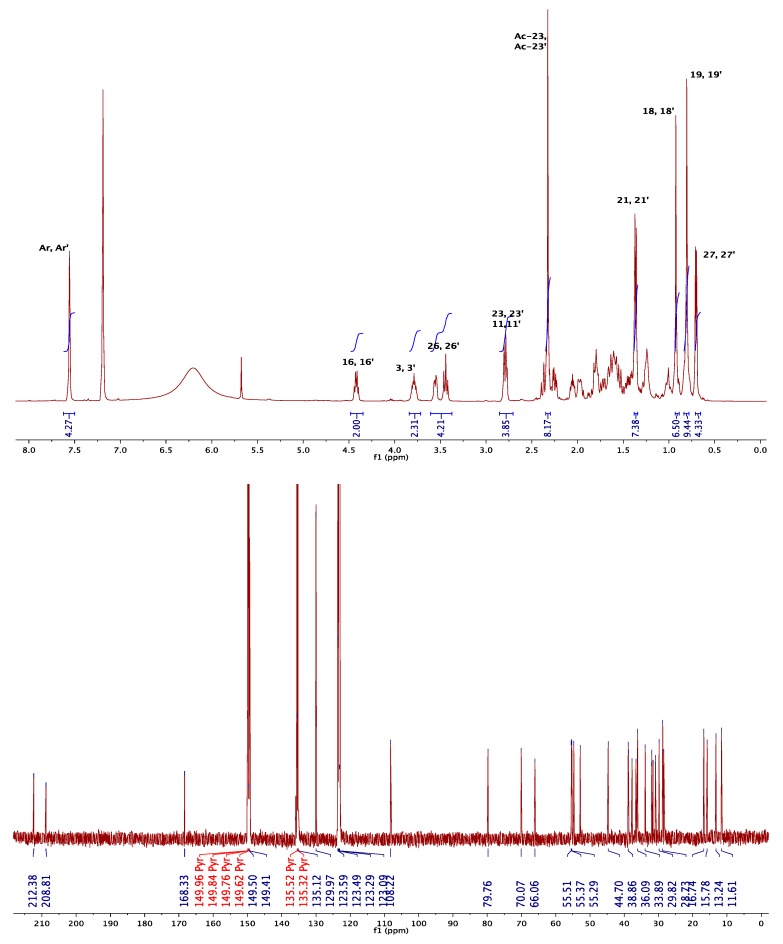
^1^H-NMR spectrum at 500 MHz in pyridine-*d_5_* of **10b** (top); ^13^C-NMR at 125 MHz in pyridine-*d_5_* of **10b** (bottom).

Similarly, the methane H-3 is shifted to high frequencies confirming the formation of the ester at C3 and C3′ for all cases. More subtle changes in the displacements can be observed, for example the C-19 methyl protons present a slight displacement at a higher frequency (0.06 ppm **5**; 0.07 ppm **8a**; 0.07 ppm **10a**; 0.06 ppm **8b**; 0.04 **10b**, Table 3), this is due to the presence of the ester as spacer group. Although free rotation would be expected to minimize this effect, the steric hindrance generated by the steroidal moieties causes these protons to be arranged quite close to the carbonyl oxygen of terephthalate ester, leading to a shielding effect (Figure 3). In the ^1^H-NMR spectrum of **10b**, a singlet is observed at 2.35 ppm belonging to the H of the CH_3_ of position 23”; in ^13^C-NMR spectrum the signal in 208.8 ppm is assigned to the C=O group at C23” (Figure 4).

To confirm this assumption, calculations at MM2 level of theory were performed for dimeric derivatives (series 5α **6**, and 5β **8c**, Figure S34) with an allowed error of 0.001; for the 5α compound, the minimum and maximum distances between these atoms were calculated, giving a range of 4.07 Å to 5.87 Å, close enough to generate a shielding effect for methyl C-19 protons. In the case of the 5β series it goes from 4.10 to 5.74 Å, which confirms this effect, regardless of the presence or absence of the double bond in ring B. Other signals that can be affected in the same way are the protons of ring A, but due to the overlap of signals in the region 1–2 ppm it is not possible to accurately examine this effect.

**Figure 5:**
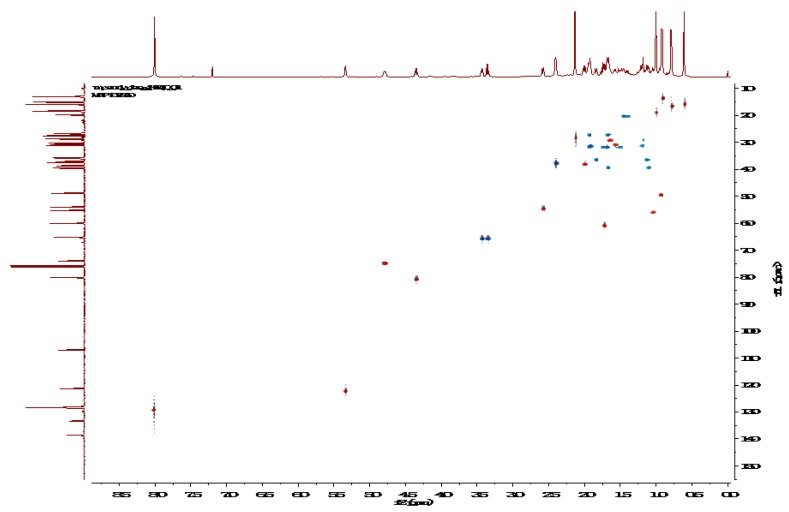
HSQC-NMR experiment at 600 MHz in CDCl_3_ of **10a**.

Two-dimensional NMR helped to see that the simple signal in 8.08 ppm belongs to the H in aromatic ring symmetrically disubstituted, that correlates with a C at 130 ppm, as observed in the HSQC spectrum of **10a** (Figure 5). Selected signals of NMR spectra of bispirostanic steroids **8a–c** and **10a–b** are shown in Table 3 and Table 4.

It should be considered that the background information of the crystal structure from X-ray diffraction can be useful to give an idea of the structure of supramolecular aggregates in aqueous solutions [43,44,45]. The crystal structures of sapogenin dimers provide information to resolve structural issues that will be subject to the structural characteristics of the steroidal framework and how it links with the spacer group (tail-to-tail or head-to-head). The most recently published example relates to the structure of a dimeric steroid with the spirostanic side chain and the open ring A in a linked tail-to-tail arrangement [5]. In addition to the last-referred paper, as far as we identify, only Bertolasi et al. [46], Bai et al. [47] Ghosh et al. [48], and Dias et al. [49] have registered crystal structures of a bile acid dimer, a bile acid cyclodimer, and a cyclotrimer derived from deoxycholate, respectively.

**Figure 6:**
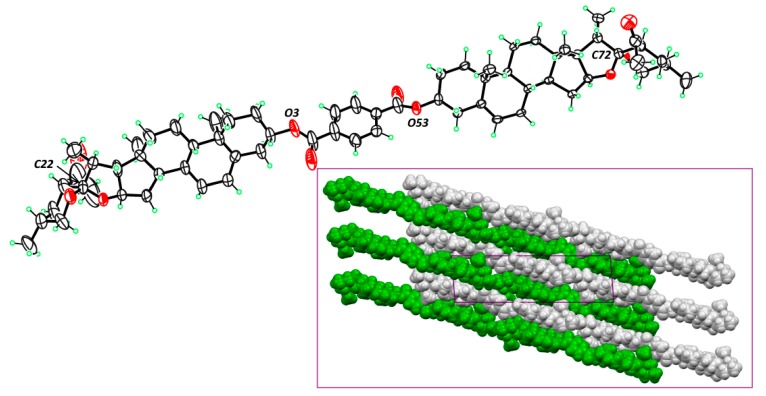
Molecular structure of the bisteroid derivative **10a**, with displacement ellipsoids at the 20% probability level. The inset shows a part of the crystal structure, viewed down crystallographic *b* axis, using a spacefill representation. Grey molecules are generated from the asymmetric unit by lattice translations, while green molecules are generated by the 2_1_-screw axis in space group *P*2_1_.

We report the crystal structure of compound **10a**, while attempts for the crystallization of other compounds were unsuccessful, perhaps because of their trend of self-assembling in solution. For the case of the compound **10a**, the single-crystal structure is in agreement with the NMR data, and shows that the central terephthalate ring is symmetrically substituted by two steroidal moieties, with the ester groups arranged in an anti-conformation (Figure 6). However, the midpoint of the central benzene ring is a non-crystallographic inversion centre, since the molecule is chiral. The full extension of the molecule is around 39 Å, and molecules are packed in the solid state in such a way that they line up in a single direction, **a–c**, see inset in Figure 6.

However, the packing structure is not efficient, as reflected in the rather low Kitaigorodskii packing index of 0.61 [50], and large voids are present, contributing to *ca* 5% of the crystal volume. Apparently, these voids are not filled by disordered solvent, since the *SQUEEZE* procedure does not improve significantly the structure refinement [51].

#### 3.1.2. Thermal Stability of Bisteroidal Esters of Terephthalic Acid

**Figure 7:**
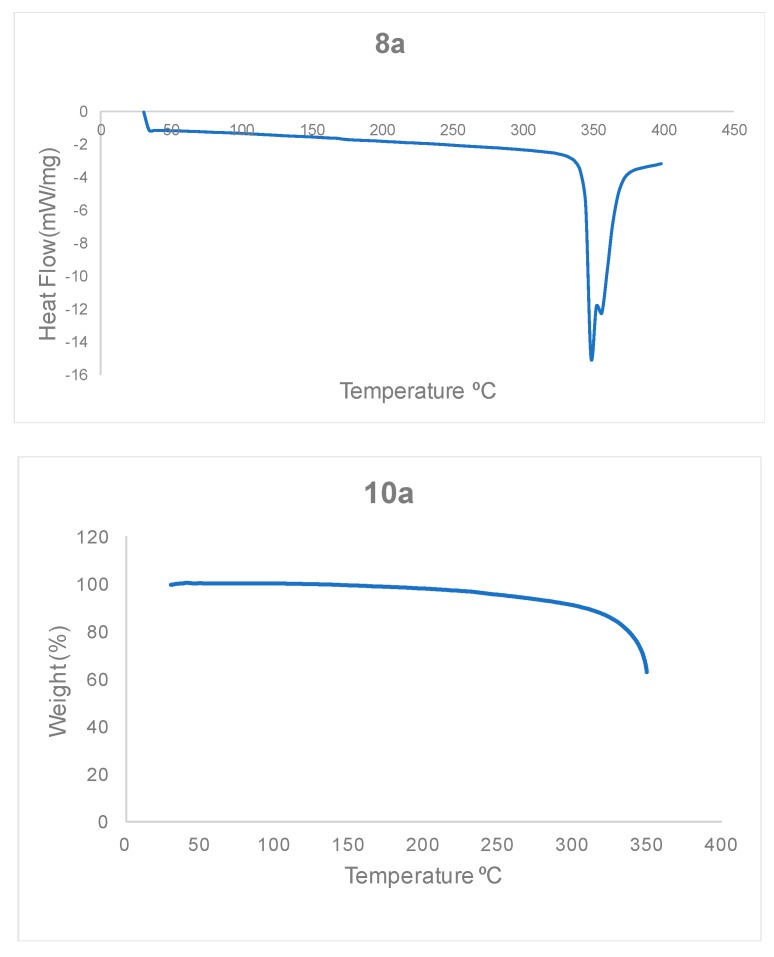
Differential scanning calorimetry (DSC, top) of **8a**. Thermogravimetric analysis (TGA, down) of **10a**.

Thermal stability analysis of bisteroids allowed to corroborate the stability of the compounds to changes in heat flux depending on temperature or time. Melting points are 317–320 °C for **8a** and 269–270 °C for **10a**, with presence of opacity. At the same time, the analyses of thermal stability for these compounds at high temperatures was evaluated using differential scanning calorimetry (DSC) and thermogravimetric analysis (TGA, Figure 7). The calorimetry studies showed that the solid samples do not contain solvent molecules within the structure and it was possible to see that the samples of the analyzed compounds are highly stable because no phase transitions were detected when heating from room temperature to 317.6 °C, where the onset of an endothermic transition was observed, reaching a peak at 320.0 °C. This endotherm was coincident with the melting of compound **8a** as it was experimentally observed. In the case of **10a** the endothermic transition was observed upon heating from room temperature up to 269.0 °C, at which point the start of an endothermic transition was observed, reaching a peak at 270.0 °C. The thermogravimetric analysis did not exhibit any loss of weight from room temperature to 350 °C, it also did not occur in any of the derivatives before reaching the melting point.

### 3.2. Responsive Self-Assembly of Bisteroidal Esters of Terephthalic Acid

For the experiments, 50 mg of the bisteroid derivative were added to a mixture of 10 mL of EtOAc and 250 mL of hexane or over pure EtOAc. The solutions were heated gently to achieve a clear and homogeneous solution, cooled to room temperature and left standing for three days. Pure ethyl acetate was chosen to study the influence of pure or mixed solvent onto the morphologies of the supramolecular structures.

**Figure 8:**
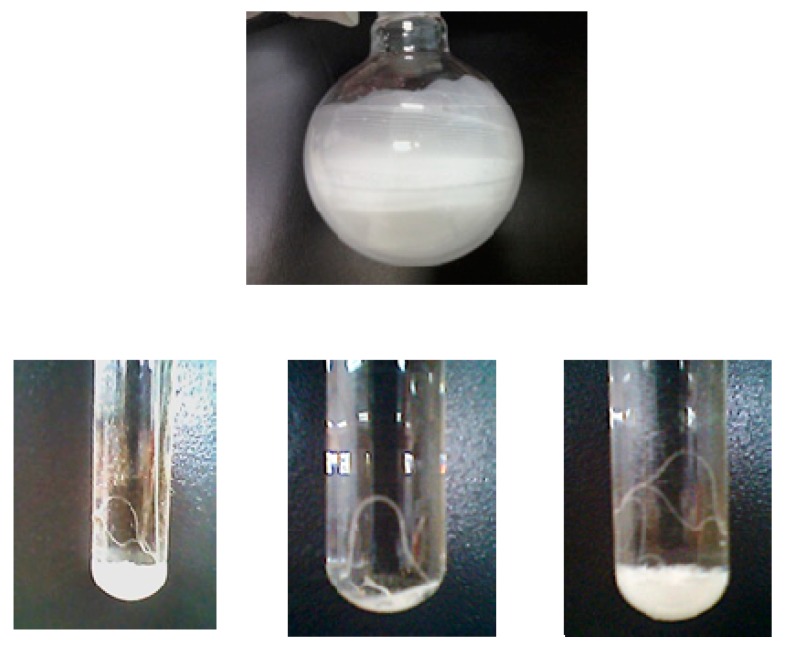
Picture of strands-shaped aggregates of derivative **8a** observed in hexane/ethyl acetate system (up) and in ethyl acetate solution (down).

**Figure 9:**
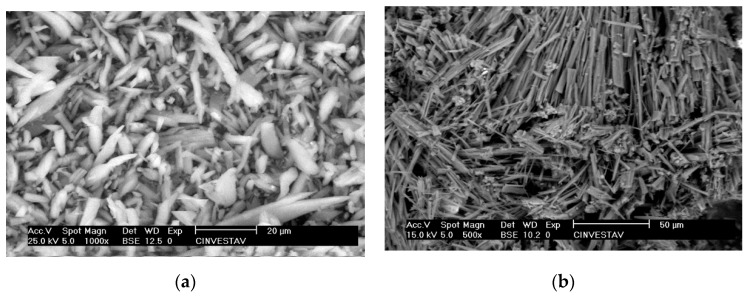
SEM images of the self-assembly strand-shaped structures of bisteroids esters: (**a**) **8a** in EtOAc. (**b**) **10b** in EtOAc.

The morphologies of bidiosgenin derivative aggregates (**8a**) were analyzed by scanning electron microscopy (SEM) to explain the supramolecular structures of such bisteroid derivatives in the self-assembly progression. In contact with hexane/ethyl acetate system, the functionalized dimer self-organized into a non-permeable, non-porous layer, since the formation of membrane-shaped aggregates of the compound was observed (Figure 8). The SEM image of the aggregates of **8a** are shown in Figure 9a. A noteworthy phenomenon was that the bisteroids show different solvent-responsive morphologies in non-polar, polar and mixture media, involving strands-shaped and membranes-shape aggregates.

In the report of Yang [31], the author worked with cholesterol, showing that the molecular structure and the solution components affect the level of aggregation. From his work, we extract that the driving force for obtaining higher-order structures is the self-assembly of the low molecular weight gelators mediated by non-covalent interactions, as van der Waals forces. This study affirmed that cholesterol keeps a rigid and planar skeleton together with the tendency to form aggregates via van der Waals interactions, which facilitate the formation of well-defined supramolecular structures.

The cholesterol conjugate can self-assemble into different fibrous structures in mixed solution or acetone through the synergistic effects between van der Waals forces of cholesterol residues and electrostatic interactions between POM groups. Yang also established that the polarity of the solvent can also be crucial and favourable for modulating supramolecular structures, where the dielectric constant ε^e^ is employed to evaluate the polarity of the solvent. He reports in his work that, in the acetone solution, van der Waals interactions between cholesterol residues are considerably reduced, because the solubility of cholesterol residues decreases significantly in acetone (ε^e^ = 20.70). In the CH_2_Cl_2_–CH_3_OH system with a dielectric constant of mixed solvents (ε^e^ = 13.02), van der Waals interactions between cholesterol residues are significantly weakened, due to the solubility of cholesterol residues in dichloromethane (ε^e^ = 9.08). That is the reason why the polarity of the solvent has an essential role in the regulation of the morphology of the self-assembly process. These results explain satisfactorily from a molecular level why the supramolecular structure of bisteroids (**5**–**6**, **8a–c**, **10a–b**) in hexane-EtOAc and pure EtOAc is better organized than that formed in CHCl_3_-CH_3_OH where the strands are thinner. From that it can be assumed that a dielectric constant of 8.2 (ε^e^ = 8.26) is ideal for obtaining better organized supramolecular structures in compounds with cholesterol residues.

**Figure 10:**
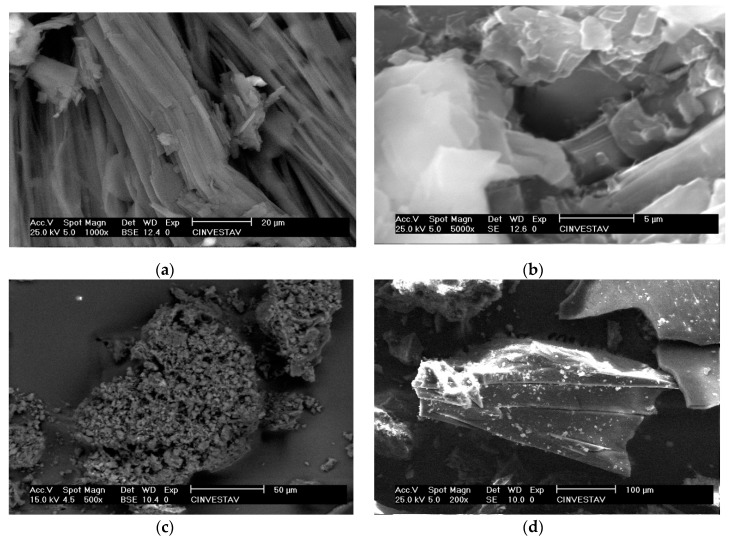
SEM images of the self-assembly structures of strand and membrane-shaped bisteroid derivatives: (**a**) **5** in EtOAc; (**b**) **5** in hexane/EtOAc (25:1) showing membrane-shaped structures; (**c**) **8c** in EtOAc (strands-shaped structures); (**d**) **8c** in hexane/EtOAc (25:1) (membrane-shaped structures).

Our results show that in the self-assembling structures of the bisteroids **5**, **6**, **8a**–**c** and **10a**–**b**, bisteroidal derivatives with self-assembled structures obtained from a hexane/EtOAc system (25:1) tend to be organized, forming membrane-shaped structures (Figure 10b,d). When these derivatives were treated with EtOAc, the bisteroid esters were self-organized in a strand-shape with a length greater than 100 µm (Figure 10a,c; see Figures S42–S46 in SM). Since the solubility increases in chloroform, van de Waals interactions between bisteroid moieties are weakened. As a consequence, thinner strands are formed in the CHCl_3_/CH_3_OH system (1:25) (see Figures S39 and S40 in SM). These morphologies are consistent with the values of the dielectric constant of the solvent systems, confirming that a solvent with a dielectric constant near to 8.2 (Tol-DMF ε^e^ = 8.26 [31]) will present better-organized self-assembled structures. From those results, we could observe that EtOAc (ε^e^ = 6.0) is the solvent that leads bisteroids towards better-organized self-assemblies. The scope of the self-assembly scale extends to a length close to 100 μm, at least for **8a** (see Figure S40 in SM).

Given that bisteroids are rigid and almost linear molecules, with a dimension approaching 40 Å, the oriented aggregation of these molecules in suitable solvent mixtures seems to be favoured, through alignment by physical forces, for example by van der Waals interactions [52]. Once such mesoscale assemblies have been stabilized, a mesocrystal can be formed, which is extended over much larger dimensions, in the range 10–100 μm, as observed by SEM.

**Figure 11:**
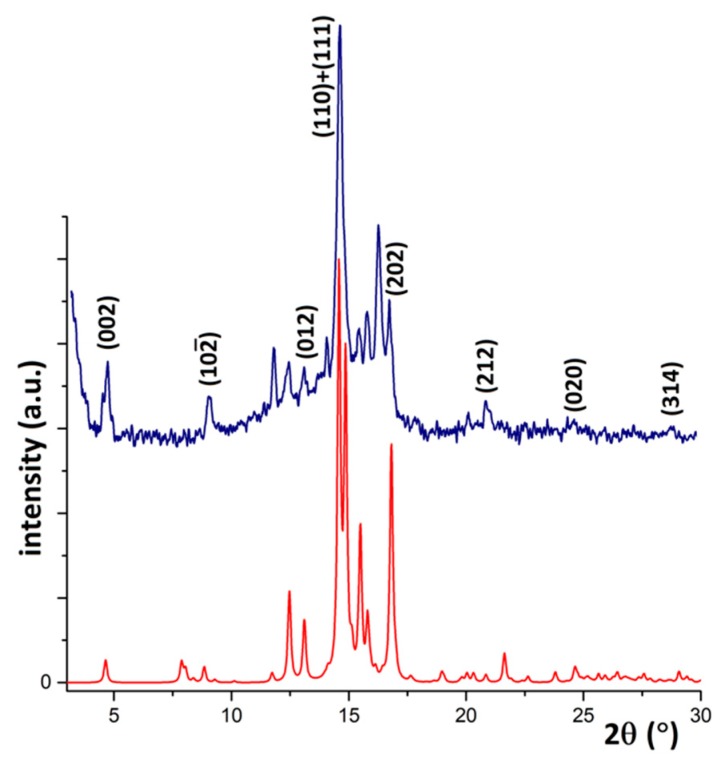
Top (blue pattern): experimental p-XRD pattern for compound **10a**, after baseline correction, with indexation of main planes. Bottom (red pattern): simulated p-XRD pattern for **10a** [55] based on the single-crystal structure (Figure 6), assuming the presence of preferred orientation. Main simulation parameters are λ = 1.54056 Å, *r* = 1.8, FWHM = 0.15° (2θ scale), and preferred orientation = axis *c.*

The self-organization description of these materials can also be indirectly averaged over much larger scales, by means of powder X-ray diffraction (p-XRD). A thin layer of compound **10a**, for which the single-crystal structure refinement allows the calculation of diffraction patterns, was characterized by p-XRD, employing standard experimental conditions (λ = 1.5406 Å). On the other hand, simulated spectra were calculated, considering the March-Dollase approach for a non-randomly oriented sample [53]. The intensity ratio *I*_111_/*I*_202_ ≈ 0.5 observed experimentally was used as a guide during pattern simulation, affording an optimized March-Dollase parameter *r* = 1.8 and a preferred orientation along axis *c*, as shown in Figure 11. The parameter *r* > 1 indicates an essentially needle-like texture, consistent with SEM images. A rough estimation of the degree of preferred orientation can be computed as *η* = [(1-*r*)^3^/(1-*r*^3^)]^1/2^, as suggested by Zolotoyabko [54]. Based on the simulated pattern of Figure 11, a degree of preferred orientation of 33% is calculated for **10a**, reflecting the spatial self-organization of this material.

## 4. Material and Methods

### 4.1. General Procedures and Materials

^1^H-and ^13^C-NMR along with DEPT, COSY, HMQC, and HMBC experiments were recorded on an NMR Bruker AVANCE Spectrometer (500 MHz for ^1^H, 125 MHz for ^13^C; Bruker Corporation, MA 01821, USA) and an Agilent NMR Spectrometer (600 MHz for ^1^H, 150 MHz for ^13^C; Agilent, Santa Clara, CA 95051, USA). Chemical shifts are stated in ppm (δ), and are referred to the residual ^1^H signal (δ = 7.27 ppm) or to the central ^13^C triplet signal (δ = 77.0 ppm) for CDCl_3_ (Sigma Aldrich, MO, USA); in pyridine-*d_5_* (Sigma Aldrich, MO, USA) the residual ^1^H signals (δ = 7.19, 7.55, 8.71 ppm) or to the ^13^C signals (δ = 123.5, 135.5, 149.5 ppm). Coupling constants (*J*) are expressed in Hz. All assignments were confirmed with the aid of two-dimensional experiments (COSY, HSQC, and HMBC). The NMR data were processed using MestreNova 12.0 software (Mestrelab Research, Spain). IR spectra were acquired on a Perkin Elmer Frontier apparatus (ν, cm^−1^) (PerkinElmer Inc., Waltham, MA, USA). High-resolution mass spectra were obtained by the fast atom bombardment (FAB) technique, using a JEOL The MStation spectrometer (JEOL USA, Inc., Peabody, MA 01960, USA). Optical rotations were determined on a Perkin Elmer 241 polarimeter (PerkinElmer Inc., Waltham, MA, USA) at room temperature using chloroform and ethanol solutions. Melting points were obtained from a Mel-Temp apparatus (Cole-Parmer, Beacon Road, Stone, Staffordshire, ST15 OSA, UK) and are uncorrected. Analytical TLC was performed on silica gel precoated ALUGRAM^®^ SIL G/UV254 (Macherey-Nagel™) plates stained with a 50% aqueous solution of H_2_SO_4_. Column chromatography was carried out employing silica gel Davisil^TM^ grade 633 (200–425 mesh, Sigma Aldrich, MO, USA).

Single-crystal X-ray diffraction data of the dispirostene ester **10a** were collected at room temperature with an Xcalibur diffractometer (Oxford Diffraction, Yarnton, UK; CrysalisPro 1.171)) equipped with an Atlas detector and an Enhance X-ray source (CuKα radiation, λ = 1.5418 Å). The refinement and structure factors have been deposited with the CCDC (Deposition code: CCDC-1951523).

#### 4.1.1. Scanning Electron Microscopy (SEM)

The morphology analysis of the self-assembled bisteroidal esters was performed on a Philips XL30 ESEM microscope. SEM samples were prepared by coating a gold film on the samples. The metal was applied in a controlled manner in a sputter coater.

#### 4.1.2. Differential Scanning Calorimetry (DSC)

Molar fractions were measured through Differential Scanning Calorimetry (DSC), DSC Mettler Toledo Star 1 device (Mettler-Toledo GmbH, Analytical, CH-8603 Schwerzenbach, Switzerland), and estimated purities were above 0.98. Subsequently, samples were placed into hermetically sealed aluminium cells of 40 µL, and heated under a constant flow of dry nitrogen atmosphere with a heating rate of 10 ° C/min.

#### 4.1.3. Thermogravimetry/TGA

Thermogravimetric analysis was obtained on a Perkin Elmer Diamond Thermogravimetric device (PerkinElmer Inc., Waltham, MA, USA) under argon atmosphere using a temperature interval from 25 to 350 °C with a heating rate of 10 °C/min.

#### 4.1.4. Powder X-ray Diffraction

Powder diffraction patterns were collected on a Bruker D-8 Advance diffractometer (Bruker, Madison, WI, USA; Diffrac-Eva 4.3) (Cu-Kα radiation, 40 kV, 30 mA), over the 2θ range 5–60°, with a step size of 0.02° (0.5 s per step). Samples were mounted on a silicon holder.

### 4.2. Chemical Synthesis and Characterization

#### 4.2.1. Preparation and Structural Characterization of Bisteroidal Esters of Terephthalic Acid. General Procedure

In a 125 mL round bottom flask, the sapogenin **7a–c**, or **9a–b** (3 g, 7.2 mmol) was dissolved in pyridine (30 mL); then, terephthaloyl chloride (0.6 g, 10.8 mmol) was added. The reaction mixture was magnetically stirred at 115 °C for 3 h. After that, the mixture was cooled down. The organic phase was extracted with DCM (25 mL × 3), washed with brine (2 × 20 mL), followed by neutralization with solution of 5% HCl. The crude product was dried over anhydrous Na_2_SO_4_ and concentrated to dryness under vacuum. The resulting bisteroids were purified by solvent partitioning methods from crude. Bispirostanes **8a–c** and **10a–b** were obtained as colorless solids. The same procedure was applied for obtaining bicholestanol ester (**5**) and bicholesterol ester (**6**) starting from (**3**) or (**4**), respectively.

#### 4.2.2. Bicholesteryl Terephthalate (**5**)

Yield (83%); mp 258–260 °C (hexane/EtOAc); [α]_D_ +33° (c = 0.0011). IR cm^−1^: 2931 (CH_3_–, –CH_2_–); 1715 (C=O-O terephthalate), 1271, 1080 (C–O–C). HRMS (FAB) *m*/*z* for C_62_H_94_O_4_ Calcd: 903.4574; obs: 903.4568. ^1^H NMR δ: 8.08 (s, 4H, H-phenylene), 5.45 (2H, m, H-6, H-6′), 5.10 (2H, m, H-3, H-3′), 2.29 (4H, m, H-4, H-4′), 1.09 (2H, m, H-17-H-17′) 1.00 (6H, s, CH_3_-19, CH_3_-19′), 0.91 (6H, d, J_21-20_ = 7.2 Hz, CH_3_-21, CH_3_-21′), 0.86 (12H, s, CH_3_-26, CH_3_-26′), 0.68 (6H, d, J_27-25_ = 6.4 Hz, CH_3_-18- CH_3_-18′). ^13^C NMR δ: 37.2 (C-1, C-1′), 31.4 (C-2, C-2′), 74.8 (C-3, C-3′), 41.4 (C-4, C-4′), 140.8 (C-5, C-5′), 121.4 (C-6, C-6′), 50.0 (C-9, C-9′), 36.6 (C-10, C-10′), 20.8 (C-11, C-11′), 39.7 (C-12, C-12′), 40.2 (C-13, C-13′), 56.5 (C-14, C-14′), 31.8 (C- 15, C-15′), 80.8 (C-16, C-16′), 62.0 (C-17, C-17′), 16.3 (C-18, C-18′), 19.4 (C-19, C-19′), 41.6 (C-20, C-20′), 14.5 (C-21, C-21′), 31.3 (C-23, C-23′), 28.7 (C- 24, C-24′), 30.3 (C-25, C-25′), 28.5 (C-26, C-26′), 17.1 (C-27, C-27′), 129.5 (CH phenylene); 134.3 (C-ipso 1, 4; 1′, 4′) 165.25 (COO-3, COO-3′).

#### 4.2.3. Bicholestan-3-yl Terephthalate (**6**)

Yield (83%); mp 254–256 °C (hexane/EtOAc); [α]_D_ +6° (c = 0.0014). IR cm^−1^: 2930 (CH_3_–, –CH_2_–); 1716 (C=O-O terephthalate), 1272, 1114 (C–O–C). HRMS (FAB) *m*/*z* for C_62_H_98_O_4_ Calcd: 907.4620; obs: 907.4658. ^1^H NMR δ: 8.08, (4H, s, H-phenylene), 4.96 (2H, ddt, J_3-2a_ = 16.15 Hz, J_3-4a_ = 11.35 Hz, J_3-2e_ = J_3-4e_ = 4.88 Hz, H-3, H-3′) 0.90 (6H, d, J_3-2a_ = 6.39 Hz, CH_3_-21, CH_3_-21′), 0.87 (6H, s, CH_3_-18, CH_3_-18′), 0.87 y 0.86 (6H, d, J_3-2a_ = 2.56, CH_3_-26 y CH_3_-26′), 0.66 (6H, s, CH_3_-19, CH_3_-19′). ^13^C NMR δ: 165.40 (COO-3, COO-3′), 131.3 (C-ipso 1, 4; 1′, 4′), 129.4 (CH-phenylene), 74.9 (C-3, C-3′) 56.3 (C-17, C-17′), 56.2 (C-16, C-16′), 54.2 (C-5, C-5′), 44.6 (C-14, C-14′), 42.6 (C-11, C-11′), 39.5 (C-20, C-20′), 32.0 (C-1, C-1′) 31.5 (C-15, C15′), 28.6 (C-4, C-4′), 18.7 (C-21, C-21′), 12.3 (C-18, C-18′), 12.1 (C-19, C-19′).

#### 4.2.4. Bidiosgeninyl 1,4-Terephthalate (**8a**)

Yield (85%); mp 317–320 °C (hexane/EtOAc); [α]_D_ -67.71° (c = 0.0022). IR cm^−1^: 2942 (CH_3_–, –CH_2_–); 1713 (C=O-O terephthalate), 1274, 1116 (C–O–C). HRMS (FAB) *m*/*z* for C_62_H_86_O_8_ Calcd: 959.6401; obs: 959.6414. ^1^H NMR (600 MHz) δ: 8.08 (s, 4H, H-phenylene), 5.44 (2H, d, J = 5.2 Hz, H-6, H-6′), 4.91 (2H, ma, H-3, H-3′), 4.43 (2H, ddd, J_16-15a_ = J_16-15e_ = J_16-17_ = 7.6 Hz, H-16, H-16′), 3.48 (2H, ddd, J_gem_ = 10.8, J_26e- 25a_ = 4.4 J_26e-24e_ = 2.4 Hz, H-26e, H-26e’), 3.38 (2H, dd, J_gem_ = J_26a-25a_ = 10.8Hz, H-26a, H- 26a’), 2.50 (4H, d, J_4a-3_ = 8 Hz, H-4, H-4′), 2.0 (2H, d, J_17-16_ =7.6 Hz, H-17-H-17′) 1.10 (6H, s, CH_3_-19, CH_3_-19′), 0.99 (6H, d, J_21-20_ = 7.2 Hz, CH_3_-21, CH_3_-21′), 0.80 (6H, s, CH_3_-18, CH_3_-18′), 0.79 (6H, d, J_27-25_ = 6.4 Hz, CH_3_-27- CH_3_-27′). ^13^C NMR (150 MHz) δ: 37.2 (C-1, C-1′), 31.4 (C-2, C-2′), 75.2 (C-3, C-3′), 41.4 (C-4, C-4′), 140.8 (C-5, C-5′), 121.4 (C-6, C-6′), 32.0 (C-7, C-7′), 31.6 (C-8, C-8′), 50.0 (C-9, C-9′), 36.6 (C-10, C-10′), 20.8 (C-11, C-11′), 39.7 (C-12, C-12′), 40.2 (C-13, C-13′), 56.4 (C-14, C-14′), 31.8 (C- 15, C-15′), 81.0 (C-16, C-16′), 62.0 (C-17, C-17′), 16.3 (C-18, C-18′), 19.4 (C-19, C-19′), 41.6 (C-20, C-20′), 14.5 (C-21, C-21′), 109.5 (C-22, C-22′), 31.3 (C-23, C-23′), 28.7 (C- 24, C-24′), 30.3 (C-25, C-25′), 66.9 (C-26, C-26′), 17.1 (C-27, C-27′), 129.7 (CH-phenylene); 134.4 (C-ipso 1, 4; 1′, 4′), 165.4 (COO-3, COO-3′).

#### 4.2.5. Bihecogeninyl Terephthalate (**8b**)

Yield (88%); mp 261–262 °C (hexane/EtOAc); [α]_D_ -6.62° (c = 0.0018). IR cm^−1^: 2928 (CH_3_–, –CH_2_–); 1702 (C=O-O terephthalate), 1672 (stretching C=O C-), 1279, 1038 (C–O–C). HRMS (FAB) *m*/*z* for C_62_H_86_O_10_ Calcd: 990.6621; obs: 990.6619. NMR ^1^H δ: 8.06 (s, 4H, Ar), 4.95(2H, tt, J_3-2a_ = J_3-4a_ = 11.0 Hz, J_3-2e_ = J_3-4e_ = 4.7 Hz, H-3, H-3′), 4.34 (2H, ddd, J_16-15a_ = 8.8 Hz, J_16-15e_ = 7.4 Hz, J_16-17_ = 6.2 Hz, H-16, H-16′), 3.50 (2H, m, H-26e, H-26e’), 3.35 (2H, dd, J_gem_ = J_26a-25a_ = 10.6Hz, H-26a, H- 26a’), 2.53 (2H, dd, J_gem_ = 14.2 Hz, J_11a-10_ = 13.2 Hz, H-11a, H-11a’), 2.42 (2H, dd, J_gem_ = 14.2 Hz, J_11e-10_ = 5.0 Hz, H-11e, H-11e’), 1.08 (6H, d, J_21-20_ = 7.2 Hz, CH_3_-21, CH_3_-21′), 1.06 (6H, s, CH_3_-18, CH_3_-18′), 0.98 (6H, s, CH_3_-19, CH_3_-19′), 0.78 (6H, d, J_27-25_ = 6.4 Hz, CH_3_-27- CH_3_-27′). ^13^C NMR δ: 37.2 (C-1, C-1′), 31.4 (C-2, C-2′), 74.3 (C-3, C-3′), 41.4 (C-4, C-4′), 31.3 (C-7, C-7′), 30.1 (C-8, C-8′), 50.0 (C-9, C-9′), 36.1 (C-10, C-10′), 20.8 (C-11, C-11′), 213.3 (C-12, C-12′), 40.2 (C-13, C-13′), 39.3 (C-14, C-14′), 31.4 (C- 15, C-15′), 79.1 (C-16, C-16′), 66.8 (C-17, C-17′), 16.0 (C-18, C-18′), 17.1 (C-19, C-19′), 41.6 (C-20, C-20′), 13.2 (C-21, C-21′), 109.2 (C-22, C-22′), 31.4 (C-23, C-23′), 28.7 (C- 24, C-24′), 30.1 (C-25, C-25′), 66.8 (C-26, C-26′), 11.9 (C-27, C-27′), 130.0 (CH-phenylene); 134.3 (C-ipso 1, 4; 1′, 4′), 165.2 (COO-3, COO-3′).

#### 4.2.6. Bisarsasapogeninyl Terephthalate (**8c**)

Yield (80%), mp 204–206 °C (hexane/EtOAc); [α]_D_ -23.33° (c = 0.0018). IR cm^−1^: 2929 (CH_3_–, –CH_2_–); 1718 (C=O-O terephthalate), 1269, 1117 (C–O–C). HRMS (FAB) *m*/*z* for C_62_H_90_O_8_ Calcd: 962.6636; obs: 962.6619. ^1^H NMR δ: 8.11 (s, 4H, Ar), 5.36 (2H, m, H-3, H-3′), 4.42 (2H, m, H-16, H-16′), 3.95 and 3.31 (4H, d, J_26e- 25a_ = 4.4 H-26, H-26′), 1.09 (6H, d, J_21-20_ = 7.2 Hz, CH_3_-27, CH_3_-27′), 1.04 (6H, s, CH_3_-19, CH_3_-19′), 1.0 (6H, d, J_21-20_ = 7.2 Hz, CH_3_-21, CH_3_-21′), 0.78 (6H, s, CH_3_-18, CH_3_-18′). ^13^C NMR δ: 37.7 (C-1, C-1′), 31.4 (C-2, C-2′), 71.9 (C-3, C-3′), 40.0 (C-4, C-4′), 38.5 (C-5, C-5′), 28.5 (C-6, C-6′), 32.0 (C-7, C-7′), 31.6 (C-8, C-8′), 42.1 (C-9, C-9′), 36.6 (C-10, C-10′), 20.9 (C-11, C-11′), 40.2 (C-12, C-12′), 40.6 (C-13, C-13′), 56.3 (C-14, C-14′), 31.8 (C- 15, C-15′), 80.9 (C-16, C-16′), 62.0 (C-17, C-17′), 16.0 (C-18, C-18′), 24.0 (C-19, C-19′), 41.6 (C-20, C-20′), 14.3 (C-21, C-21′), 109.7 (C-22, C-22′), 30.3 (C-25, C-25′), 65.1 (C-26, C-26′), 16.5 (C-27, C-27′), 129.4 (CH-phenylene); 134.7 (C-ipso 1, 4; 1′, 4′) 165.1 (COO-3, COO-3′).

#### 4.2.7. Bi-(23R)-23-acetyldiosgeninyl Terephthalate (**10a**)

Yield (85%); mp 269–270 °C (hexane/EtOAc), [α]_D_ -67.93° (c = 0.0014). IR cm^−1^: 2949 (CH_3_–, –CH_2_–); 1715 (C=O-O terephthalate), 1695 (stretching C=O C-), 1269, 1119 (C–O–C). HRMS (FAB) *m*/*z* for C_66_H_90_O_10_ Calcd: 1043.6612; obs: 1043.6609. ^1^H NMR δ: 8.08 (s, 4H, Ar), 5.42 (2H, d, J = 5.2 Hz, H-6, H-6′), 4.88 (2H, ma, H-3, H-3′), 4.44 (2H, ddd, J_16-15a_ = J_16-15e_ = J_16-17_ = 7.6 Hz, H-16, H-16′), 3.50 (2H, ddd, J_gem_ = 10.8, J_26e- 25a_ = 4.4 J_26e-24e_ = 2.4 Hz, H-26e, H-26e’), 3.43 (2H, dd, J_gem_ = J_26a-25a_ = 10.8Hz, H-26a, H- 26a’), 2.65 (2H, d, J_17-16_ =7.6 Hz, H-17-H-17′), 2.46 (4H, d, J_4a-3_ = 8 Hz, H-4, H-4′), 2.21 (6H, m, CH_3_CO-23), 1.07 (6H, s, CH_3_-19, CH_3_-19′), 0.98 (6H, d, J_21-20_ = 7.2 Hz, CH_3_-21, CH_3_-21′), 0.87 (6H, s, CH_3_-18, CH_3_-18′), 0.68 (6H, d, J_27-25_ = 6.4 Hz, CH_3_-27- CH_3_-27′). ^13^C NMR δ: 37.2 (C-1, C-1′), 31.4 (C-2, C-2′), 75.2 (C-3, C-3′), 38.5 (C-4, C-4′), 139.6 (C-5, C-5′), 122.5 (C-6, C-6′), 32.0 (C-7, C-7′), 31.6 (C-8, C-8′), 55.1 (C-9, C-9′), 36.8 (C-10, C-10′), 20.8 (C-11, C-11′), 39.7 (C-12, C-12′), 49.9 (C-13, C-13′), 61.25 (C-14, C-14′), 31.8 (C- 15, C-15′), 81.2 (C-16, C-16′), 56.4 (C-17, C-17′), 16.2 (C-18, C-18′), 19.4 (C-19, C-19′), 41.6 (C-20, C-20′), 14.2 (C-21, C-21′), 108.1 (C-22, C-22′), 29.8 (C-23, C-23′), 28.7 (C- 24, C-24′), 31.3 (C-25, C-25′), 66.2 (C-26, C-26′), 17.0 (C-27, C-27′), 129.5 (CH-phenylene); 134.4 (C-ipso 1, 4; 1′, 4′), 165.3 (COO-3, COO-3′), 210.4 (CH_3_CO-23, CH_3_CO-23′).

#### 4.2.8. Bi-(23R)-acetylhecogeninyl Terephthalate (**10b**)

Yield 80%; mp 240–241 °C (hexane/EtOAc), [α]_D_ -2° (c = 0.0013, methanol). IR cm^−1^: 2957 (CH_3_–, –CH_2_–); 1701 (C=O-O terephthalate), 1685 (stretching C=O C-), 1280, 1045 (C–O–C). HRMS (FAB) *m*/*z* for C_66_H_90_O_12_ Calcd:1074.4136; obs:1074.4144. ^1^H NMR (Py-d_5_) δ: 7.56 (s, 4H, Ar), 4.44 (2H, ddd, J_16-15a_ = 8.8 Hz, J_16-15e_ = 7.4 Hz, J_16-17_ = 6.2 Hz, H-16, H-16′), 3.81 (2H, tt, J_3-2a_ = J_3-4a_ = 11.0 Hz, J_3-2e_ = J_3-4e_ = 4.7 Hz, H-3, H-3′), 3.54 (2H, m, H-26e, H-26e’), 3.44 (2H, dd, J_gem_ = J_26a-25a_ = 10.6Hz, H-26a, H- 26a’), 2.78 (2H, m, H-23, H-23′), 2.33 (6H, m, CH_3_CO-23), 2.27 (2H, dd, J_gem_ = 14.2 Hz, J_11e-10_ = 5.0 Hz, H-11e, H-11e’), 1.37 (6H, d, J_21-20_ = 7.2 Hz, CH_3_-21, CH_3_-21′), 0.90 (6H, s, CH_3_-18, CH_3_-18′), 0.82 (6H, s, CH_3_-19, CH_3_-19′), 0.71 (6H, d, J_27-25_ = 6.4 Hz, CH_3_-27- CH_3_-27′). ^13^C NMR (Py-d_5_) δ: 30.9 (C-1, C-1′), 33.1 (C-2, C-2′), 70.0 (C-3, C-3′), 38.8 (C-4, C-4′), 29.8 (C-5, C-5′), 28.7 (C-6, C-6′), 32.0 (C-7, C-7′), 31.6 (C-8, C-8′), 55.2 (C-9, C-9′), 37.2 (C-10, C-10′), 20.8 (C-11, C-11′), 212.3 (C-12, C-12′), 40.2 (C-13, C-13′), 56.4 (C-14, C-14′), 31.8 (C- 15, C-15′), 79.7 (C-16, C-16′), 55.3 (C-17, C-17′), 12.7 (C-18, C-18′), 16.9 (C-19, C-19′), 54.7 (C-20, C-20′), 14.3 (C-21, C-21′), 108.2 (C-22, C-22′), 29.8 (C-23, C-23′), 44.7 (C-24, C-24′), 30.3 (C-25, C-25′), 66.0 (C-26, C-26′), 17.8 (C-27, C-27′), 129.9 (CH-phenylene); 135.1 (C-ipso 1, 4; 1′, 4′), 168.3 (COO-3, COO-3′), 208.8 (CH_3_CO-23, CH_3_CO-23′).

## 5. Conclusions

In summary, the use of terephthalic acid as a linker between the steroid moieties was essential to induce the planarity of bisteroidal esters. The self-assembling behaviour of the spirostane dimers was explored. The self-organized behaviour is triggered by solvents, that leads to a non-permeable non-porous layer dimer in contact with the hexane/EtOAc system (25:1); as well as the formation of aggregates in the form of strands of the bisteroidal compounds was observed in contact with pure EtOAc and CHCl_3_/CH_3_OH system (1:25).

We conclude that non-covalent interactions (van der Waals forces) among the steroid residues of the synthesized series of symmetric bisteroids, and the polarity of the solvent systems employed might induce molecules to self-assemble into organized supramolecular structures. Our results also show that bisteroids will present different self-assembly structures depending on the characteristics of the solvent. With non-polar solvents, self-assembled structures tend to be organized into membrane-shaped structures. By the employment of polar solvents, bisteroid esters self-organized as strand-shapes. However, in the CHCl_3_/CH_3_OH system (polar solvent), thinner strands were formed because van der Waals interactions among bisteroid moieties are weaker due to the higher solubility in such system. The observed morphologies are consistent with the values of the dielectric constant of solvent systems, confirming that a solvent with a dielectric constant close to eight will present better organized self-assembled structures. In our study, EtOAc was the most effective solvent for the formation of self- assemblies.

Since the applications and importance have not yet been clearly established for this class of compounds, we plan to continue our studies in this direction.

## Figures and Tables

**Table 1:** Conditions of reaction to obtain bisteroids derivatives from cholesterol (**3**) and diosgenin (**7a**) *.

Parameter	Time	Temp	Solvent	Base	Yield
**entry**					
**a**	1 h	rt	Py	Py	nr **
**b**	2 h	rt	DMF	K_2_CO_3_	nr **
**c**	24 h	reflux	THF	Et_3_N	10%
**d**	2 h	reflux	Py	Py	70%
**e**	2 h	reflux	Py	Py	80%

* The optimized conditions are described in rows d and e. ** nr = no reaction. Entry **a**–**d** = **3** Entry **e** = **7a**.

**Table 2:** Selected ^1^H and ^13^C NMR signals for compounds **5** and **6**, in ppm.

	^1^H NMR (CDCl_3_)			^13^C NMR (CDCl_3_)	
**signals**	**5**	**6**	**signals**	**5**	**6**
H-3, H-3′	4.90	4.96	C-3, C-3′	75.1	74.9
H-18, H-18′	0.69	0.87	C-18, C-18′	18.7	12.3
H-19, H-19′	1.07	0.66	C-19, C-19′	19.3	12.0
H-21, H-21′	0.93	0.91	C-21, C-21′	11.8	18.6
H_Ar_-H_Ar_′	8.08	8.07	C_ipso_-C_ipso_′	137.1	134.4

**Table 3:** Selected ^1^H-NMR signals of the **8a–c** and **10a–b** in ppm.

Signals	8a *	8b *	8c *	10a *	10b 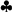
H-3, H-3′	4.91	4.95	5.36	4.80	3.81
H-6, H-6′	5.44			5.34	
H-18, H-18′	0.80	1.06	0.78	0.61	0.90
H-19, H-19′	1.10	0.98	1.04	1.00	0.82
H-21, H-21′	0.99	1.08	1.00	0.92	1.37
H-26a, H-26a′	3.38	3.35	3.31	3.36	3.44
H-26e, H-26e′	3.48	3.50	3.95	3.43	3.54
H-27, H-27′	0.79	0.78	1.09	0.80	0.71
H_Ar_-H_Ar_′	8.08	8.06	8.11	8.01	7.56

* in CDCl_3_. 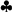 in Pyridine-*d_5_.*

**Table 4:** Selected ^13^C-NMR signals of the **8a–c** and **10a–b** in ppm.

Signals	8a *	8b *	8c *	10a *	10b 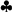
C-3, C-3′	75.2	74.3	71.9	75.2	70.0
C-5, C-5′	139.5	--	--	139.6	--
C-6, C-6′	122.7	--	--	122.5	--
C-18, C-18′	16.2	16.0	16.0	16.2	12.7
C-19, C-19′	19.3	17.1	24.0	19.4	16.9
C-21, C-21′	14.4	13.2	14.3	14.2	14.3
C-22, C-22′	109.5	109.2	109.7	108.1	108.2
C-23, C-23′	31.3	31.4	--	29.8	29.8
C-26, C-26′	66.9	66.8	65.1	66.2	66.0
C-27, C-27′	17.1	11.9	16.5	17.0	17.8
C_ipso_-C_ipso_′	134.4	134.3	134.7	134.4	135.1

* in CDCl_3_. 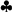 in Pyridine-*d_5_.*

## Supplementary Materials

The following are available online, Figure S1: IR spectrum of the Bicholesterol ester (**5**). Figure S2: Mass spectrum of Bicholesterol ester (**5**). Figure S3: ^13^C NMR spectrum at 125 MHz in CDCl_3_ of Bicholesterol ester (**5**). Figure S4: Differential scanning calorimetry (DSC) of Bicholesterol ester (**5**). Figure S5: ^1^H NMR spectrum at 500 MHz in CDCl_3_ of Bicholestanol ester (**6**). Figure S6: ^13^C NMR spectrum at 125 MHz in CDCl_3_ of the of Bicholestanol ester (**6**). Figure S7: HSQC-NMR spectrum at 500 MHz of Bicholestanol ester (**6**). Figure S8: IR spectrum of Bidiosgenin ester (**8a**). Figure S9: HRMS data of Bidiosgenin ester (**8a**). Figure S10: HSQC-NMR spectrum at 500 MHz in CDCl_3_ of Bidiosgenin ester (**8a**). Figure S11: Thermogravimetric analysis of Bidiosgenin ester (**8a**). Figure S12: IR spectrum of Bihecogenin ester (**8b**). Figure S13: Mass spectrum of Bihecogenin ester (**8b**). Figure S14: ^1^H-NMR spectrum at 500 MHz in CDCl3 of Bihecogenin ester (**8b**). Figure S15: ^13^C-NMR spectrum at 125 MHz in CDCl_3_ of Bihecogenin ester (**8b**). Figure S16: Differential Scanning Calorimetry analysis of Bihecogenin ester (**8b**). Figure S17: IR spectrum of Bisarsasapogenin ester (**8c**). Figure S18: ^1^H-NMR spectrum at 500 MHz in CDCl_3_ of Bisarsasapogenin ester (**8c**). Figure S19: ^13^C-NMR spectrum at 125 MHz in CDCl_3_ of Bisarsasapogenin ester (**8c**). Figure S20: HSQC-NMR spectrum at 500 MHz in CDCl_3_ of Bisarsasapogenin ester (**8c**). Figure S21: Differential Scanning Calorimetry analysis of Bisarsasapogenin ester (**8c**). Figure S22: IR spectrum of Bi-23-acetyldiosgenin ester (**10a**). Figure S23: Mass spectrum data of Bi-23-acetyldiosgenin ester (**10a**). Figure S24: ^1^H-NMR spectrum at 500 MHz in CDCl_3_ of Bi-23-acetyldiosgenin ester (**10a**). Figure S25: ^13^C-NMR spectrum at 125 MHz in CDCl_3_ of Bi-23-acetyldiosgenin ester (**10a**). Figure S26: HSQC-NMR spectrum at 500 MHz in CDCl_3_ of Bi-23-acetyldiosgenin ester (**10a**). Figure S27: HMBC-NMR spectrum in CDCl_3_ of Bi-23-acetyldiosgenin ester (**10a**). Figure S28: Differential Scanning Calorimetry analysis of Bi-23-acetyldiosgenin ester (**10a**). Figure S29: IR spectrum of Bi-23-acetylhecogenin ester (**10b**). Figure S30: Mass spectrum of Bi-23-acetylhecogenin ester (**10b**). Figure S31: HSQC-NMR spectrum at 500 MHz of Bi-23-acetylhecogenin ester (10b). Figure S32: COSY-NMR spectrum at 500 MHz of Bi-23-acetylhecogenin ester (**10b**). Figure S33: Differential Scanning Calorimetry analysis of Bi-23-acetylhecogenin ester (**10b**). Figure S34: Molecular structure with MM2 energy minimization method for **6** and **8c**. Figure S35: Bicholesterol ester (**5**) in hexane/EtOAc showing membrane-shaped structures (SEM). Figure S36: Bicholesterol ester (**5**) in hexane/ EtOAc showing membrane-shaped structures (SEM). Figure S37: Bicholesterol ester (**5**) EtOAc showing strand-shaped structures (SEM). Figure S38: Bidiosgenin ester (**8a**) in EtOAc showing strand-shaped structures (SEM). Figure S39: Bidiosgenin ester (**8a**) in CHCl_3_/MeOH (SEM). Figure S40: Bidiosgenin ester (**8a**) in CHCl_3_/MeOH (SEM). Figure S41: Bisarsasapogenin ester (**8c**) in hexane/EtOAc showing membrane-shaped structures (SEM). Figure S42: Bi-23-acetyldiosgenin ester (**10a**) in EtOAc showing strand-shaped structures (SEM). Figure S43: Bi-23-acetyldiosgenin ester (**10a**) in EtOAc showing strand-shaped structures (SEM). Figure S44: Bi-23-acetyldiosgenin ester (**10a**) in EtOAc showing strand-shaped structures (SEM). Figure S45: Bi-23-acetylhecogenin ester (**10a**) in EtOAc showing strand-shaped structures (SEM). Figure S46: Bi-23-acetylhecogenin ester (**10a**) in EtOAc showing strand-shaped structures (SEM). Figure S47: Powder X-ray diffraction patterns for raw materials of steroidal dimers **5**, **6**, **8c**, **10a** and **10b**. Patterns were collected with the Cu-Kα radiation, and are uncorrected for amorphous contribution. Figure S48: Comparison of the X-ray diffraction patterns of Bidiosgenin ester (**8a**) under different conditions: raw material, layered material, dimer in contact with chloroform-methanol, and with hexane-ethyl acetate. Patterns were collected with the Cu-Kα radiation, and are uncorrected for amorphous contribution. Note the intensity variation for the peak at 2θ = 11.5°, as a consequence of the self-organization of the steroidal dimer. CIF file of Bi-23-acetyldiosgenin (**10a**). Video: Bicholesterol ester in Hexane/EtOAc system (25:1).
