# Supplementary material for: Mesoscale Assembly of Bisteroidal Esters from Terephthalic Acid
Source: Molecules. 2020 Mar 8;25(5):1213. doi: 10.3390/molecules25051213 (PMC7179421; doi:10.3390/molecules25051213)
Supplement: Supplementary file 1 [file molecules-25-01213-s001.pdf]

Supplementary Materials

Mesoscale Assembly of Bisteroidal Esters from Terephthalic Acid

Gabriel Guerrero-Luna,[1] María Guadalupe Hernández-Linares,[2,3]* Sylvain Bernès,[4] Alan Carrasco-Carballo,[1] Diana Montalvo-Guerrero,[5] María A. Fernández-Herrera,[5] Jesús Sandoval-Ramírez.[1]

[1]Facultad de Ciencias Químicas. Benemérita Universidad Autónoma de Puebla. 72570 Puebla, Pue., México. jesus.sandoval@correo.buap.mx; gabriel.guerrero@alumno.buap.mx; alan.carballo@alumno.buap.mx

[2]Centro de Química. Instituto de Ciencias. Benemérita Universidad Autónoma de Puebla. 72570 Puebla, Pue., México. guadalupe.mghl@correo.buap.mx

[3]Laboratorio de Investigación. Herbario y Jardín Botánico Universitario. Benemérita Universidad Autónoma de Puebla. 72570 Puebla, Pue., México.

[4] Instituto de Física. Benemérita Universidad Autónoma de Puebla. 72570 Puebla, Pue., México. Sylvain_bernes@hotmail.com

[5] Departamento de Física Aplicada. Centro de Investigación y de Estudios Avanzados - Unidad Mérida, km 6 Antigua Carretera a Progreso, Cordemex, 97310 Mérida, Yuc., México. mfernandez@cinvestav.mx, diana.montalvo@cinvestav.mx

***Correspondence:** *e-mail: guadalupe.mghl@correo.buap.mx; Tel/fax 52222295500 e7039.*

Table of contents

Figures of DSC, TGA, IR, ^{1}H NMR, ^{13}C NMR, 2D-NMR and MS spectra of compounds.

Figure S1. IR spectrum of the Bicholesterol ester (**5**)……………………………….................................4

Figure S2. Mass spectrum of Bicholesterol ester (**5**)…………………………………………….................4

Figure S3. ^{13}C NMR spectrum at 125 MHz in $CDCl_3$ of Bicholesterol ester (**5**)..5

Figure S4. Differential scanning calorimetry (DSC) of Bicholesterol ester (**5**)...5

Figure S5. ^{1}H NMR spectrum at 500 MHz in $CDCl_3$ of Bicholestanol ester (**6**)...6

Figure S6. ^{13}C NMR spectrum at 125 MHz in $CDCl_3$ of the of Bicholestanol ester (**6**)................................7

Figure S7. HSQC-NMR spectrum at 500 MHz of Bicholestanol ester (**6**)……………..................................7

Figure S8. IR spectrum of Bidiosgenin ester (**8a**)..8

Figure S9. HRMS data of Bidiosgenin ester (**8a**)..8

Figure S10. HSQC-NMR spectrum at 500 MHz in $CDCl_3$ of Bidiosgenin ester (**8a**).………...……….........9

Figure S11. Thermogravimetric analysis of Bidiosgenin ester (**8a**)……………………...……...……….9

Figure S12. IR spectrum of Bihecogenin ester (**8b**)………………...10

Figure S13. Mass spectrum of Bihecogenin ester (**8b**)……...…...10

Figure S14. ^{1}H-NMR spectrum at 500 MHz in $CDCl_3$ of Bihecogenin ester (**8b**)………………….............11

Figure S15. ^{13}C-NMR spectrum at 125 MHz in $CDCl_3$ of Bihecogenin ester (**8b**)..…..................................11

Figure S16. Differential Scanning Calorimetry analysis of Bihecogenin ester (**8b**)…..................................12

Figure S17. IR spectrum of Bisarsasapogenin ester (**8c**)……...…..13

Figure S18. ^{1}H-NMR spectrum at 500 MHz in $CDCl_3$ of Bisarsasapogenin ester (**8c**)……….................13

Figure S19. ^{13}C-NMR spectrum at 125 MHz in $CDCl_3$ of Bisarsasapogenin ester (**8c**)..….......................14

Figure S20. HSQC-NMR spectrum at 500 MHz in $CDCl_3$ of Bisarsasapogenin ester (**8c**)....………..........14

Figure S21. Differential Scanning Calorimetry analysis of Bisarsasapogenin ester (**8c**)…....…................15

Figure S22. IR spectrum of Bi-23-acetyldiosgenin ester (**10a**)……………………...……………….....15

Figure S23. Mass spectrum data of Bi-23-acetyldiosgenin ester (**10a**)……………...…………...............16

Figure S24. ^{1}H-NMR spectrum at 500 MHz in $CDCl_3$ of Bi-23-acetyldiosgenin ester (**10a**)...……….......16

Figure S25. ^{13}C-NMR spectrum at 125 MHz in $CDCl_3$ of Bi-23-acetyldiosgenin ester (**10a**)…………….. 17

Figure S26. HSQC-NMR spectrum at 500 MHz in $CDCl_3$ of Bi-23-acetyldiosgenin ester (**10a**)...………...17

Figure S27. HMBC-NMR spectrum in $CDCl_3$ of Bi-23-acetyldiosgenin ester (**10a**)...……………............18

Figure S28. Differential Scanning Calorimetry analysis of Bi-23-acetyldiosgenin ester (**10a**)…………….18

Figure S29. IR spectrum of Bi-23-acetylhecogenin ester (**10b**)……………………………...………….…19

Figure S30. Mass spectrum of Bi-23-acetylhecogenin ester (**10b**)……………………………………………19

Figure S31. HSQC-NMR spectrum at 500 MHz of Bi-23-acetylhecogenin ester (**10b**)……………………..20

Figure S32. COSY-NMR spectrum at 500 MHz of Bi-23-acetylhecogenin ester (**10b**)……………………….20

Figure S33. Differential Scanning Calorimetry analysis of Bi-23-acetylhecogenin ester (**10b**)………………21

Figure S34. Molecular structure with MM2 energy minimization method for **6** and **8c**……………………21

Figures of SEM images of compounds.

Figure S35 Bicholesterol ester (**5**) in hexane/ EtOAc ……………………………………………………………22

Figure S36 Bicholesterol ester (**5**) in hexane/ EtOAc ……………………………………………………………..22

Figure S37. Bicholesterol ester (**5**) EtOAc …………………………………………………………………………23

Figure S38. Bidiosgenin ester (**8a**) in EtOAc ………………………………………………………………………..23

Figure S39. Bidiosgenin ester (**8a**) in $CHCl_3$/MeOH……………………………………………………………24

Figure S40. Bidiosgenin ester (**8a**) in $CHCl_3$/MeOH…………………………………………………………….24

Figure S41. Bisarsasapogenin ester (**8c**) in hexane/EtOAc………………………………………………………...25

Figure S42. Bi-23-acetyldiosgenin ester (**10a**) in EtOAc ……………………………………………………...25

Figure S43. Bi-23-acetyldiosgenin ester (**10a**) in EtOAc……………………………………………………….26

Figure S44. Bi-23-acetyldiosgenin ester (**10a**) in EtOAc……………………………………………………….26

Figure S45. Bi-23-acetylhecogenin ester (**10b**) in EtOAc……………………………………………………..27

Figure S46. Bi-23-acetylhecogenin ester (**10b**) in EtOAc……………………………………………………..27

Powder X-Ray Diffraction (PXRD) analysis.

Figure S47. PXRD patterns for raw materials of steroidal dimers **5**, **6**, **8c**, **10a** and **10b**…………………..28

Figure S48. Comparison of the X-ray diffraction patterns of bidiosgenin ester (**8a**) under different conditions: raw material, layered material, in contact with chloroform-methanol, and with hexane-ethyl acetate………28

Bicholesterol ester (**5**).

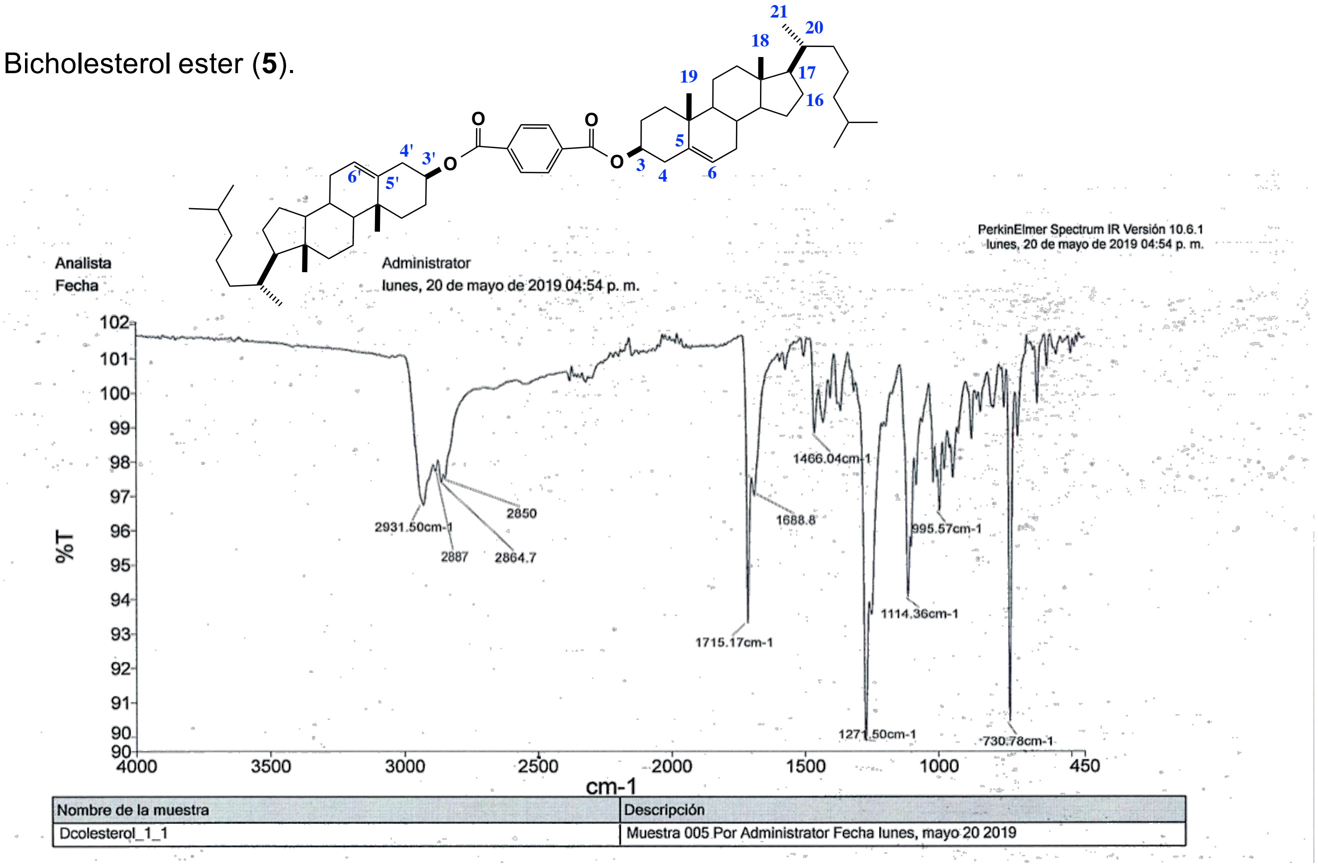

Figure S1. IR spectrum of Bicholesterol ester (**5**).

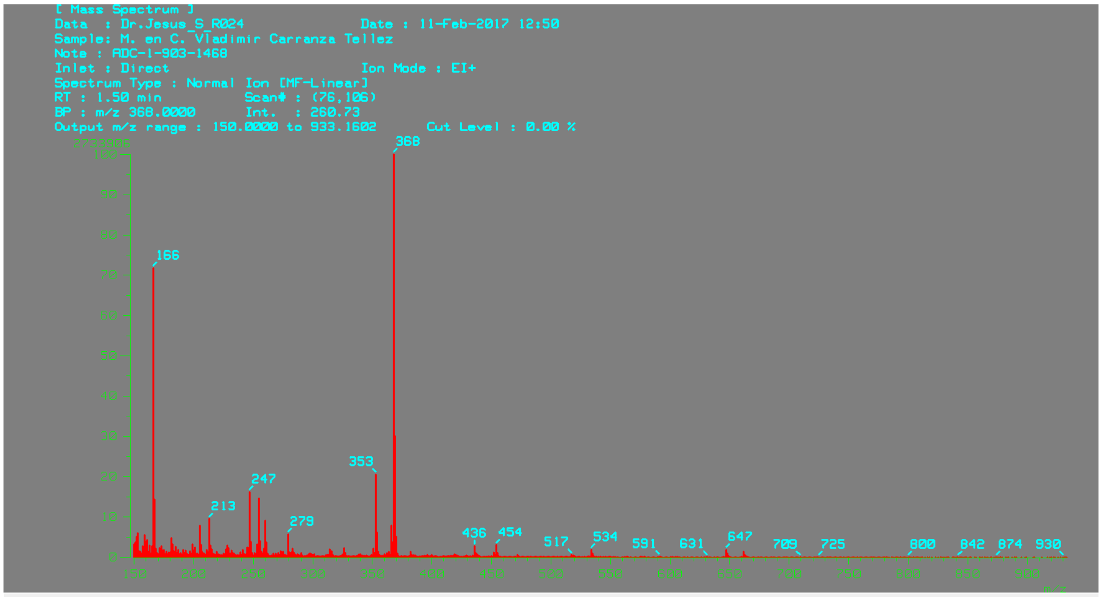

Figure S2. Mass spectrum of Bicholesterol ester (**5**).

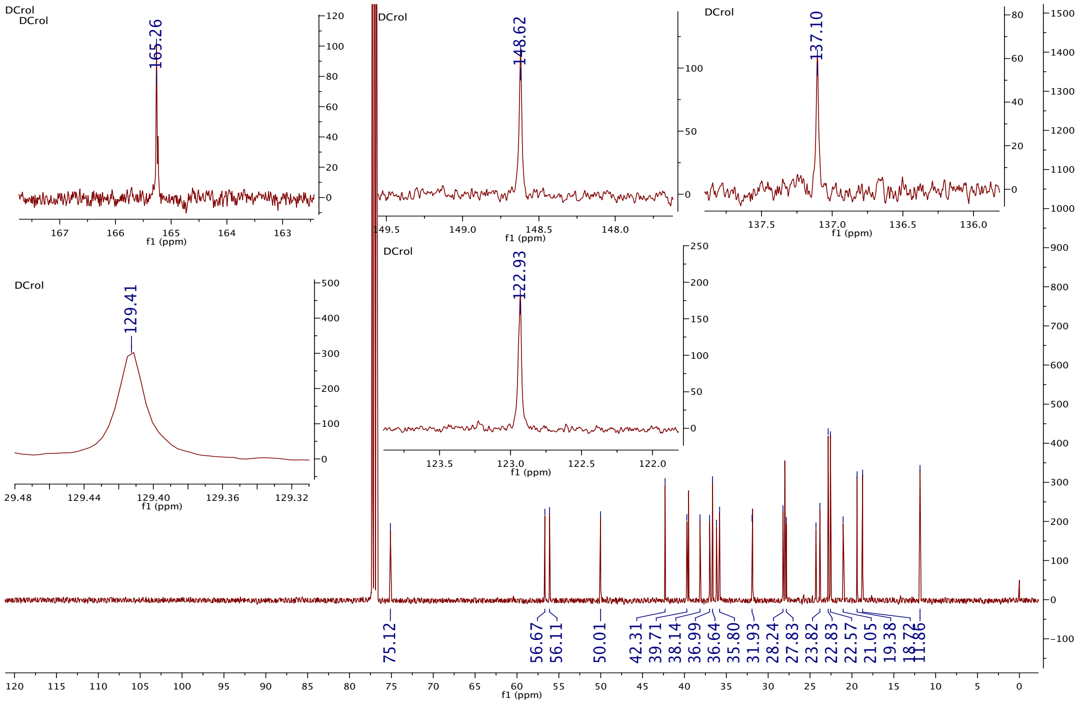

Figure S3. ^{13}C NMR spectrum at 125 MHz in $CDCl_3$ of Bicholesterol ester (**5**).

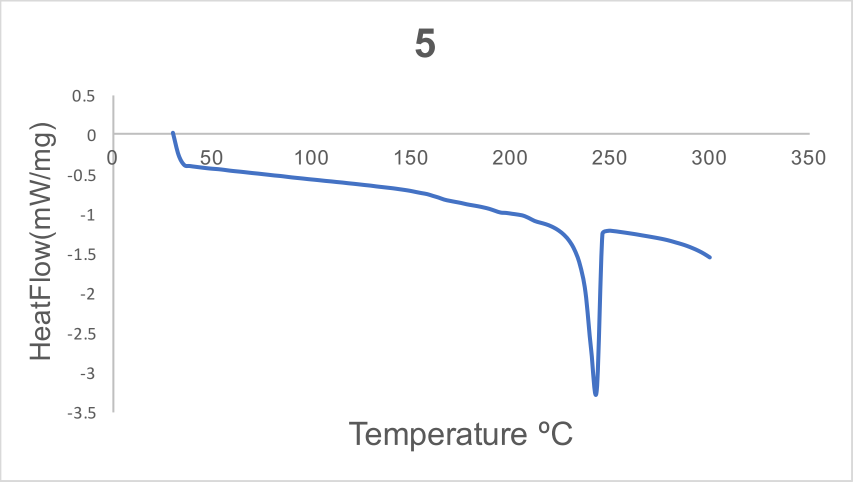

Figure S4. Differential scanning calorimetry (DSC) of Bicholesterol ester (**5**).

Bicholestanol ester (**6**)

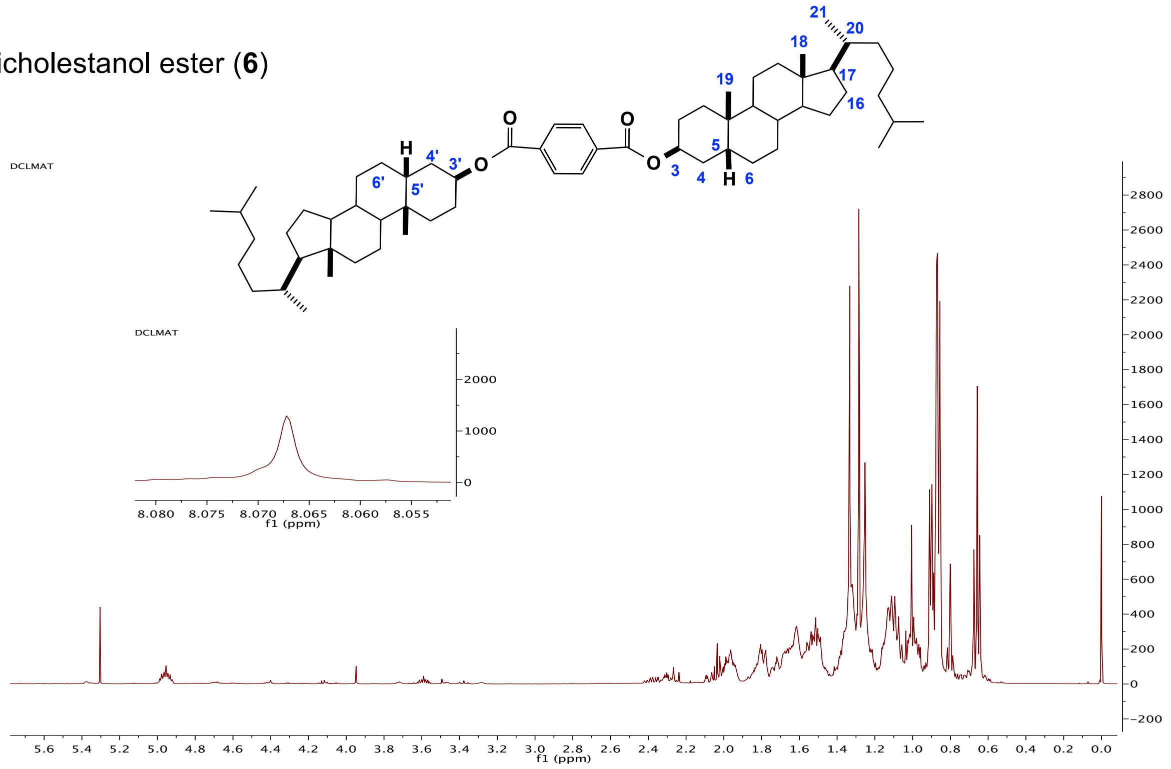

Figure S5. ^{1}H NMR spectrum at 500 MHz in $CDCl_3$ of Bicholestanol ester (**6**).

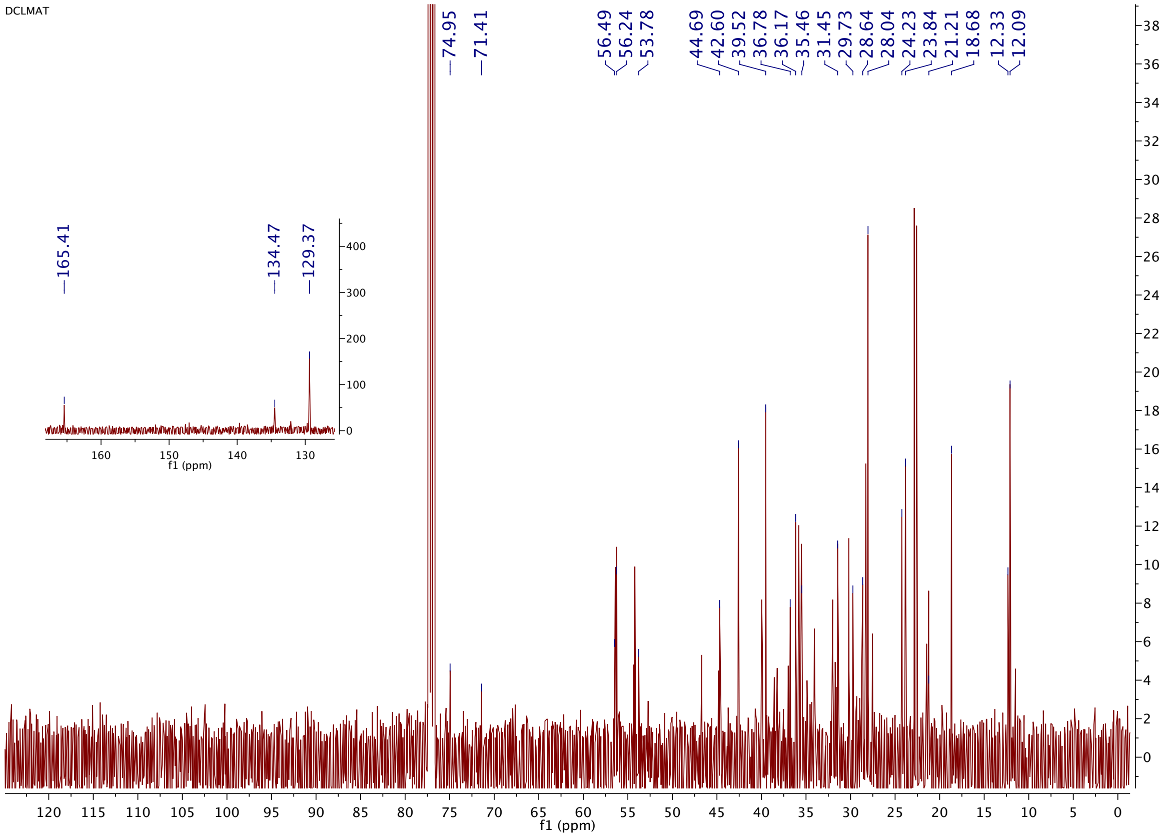

Figure S6. ^{13}C NMR spectrum at 125 MHz in $CDCl_3$ of the of Bicholestanol ester (**6**).

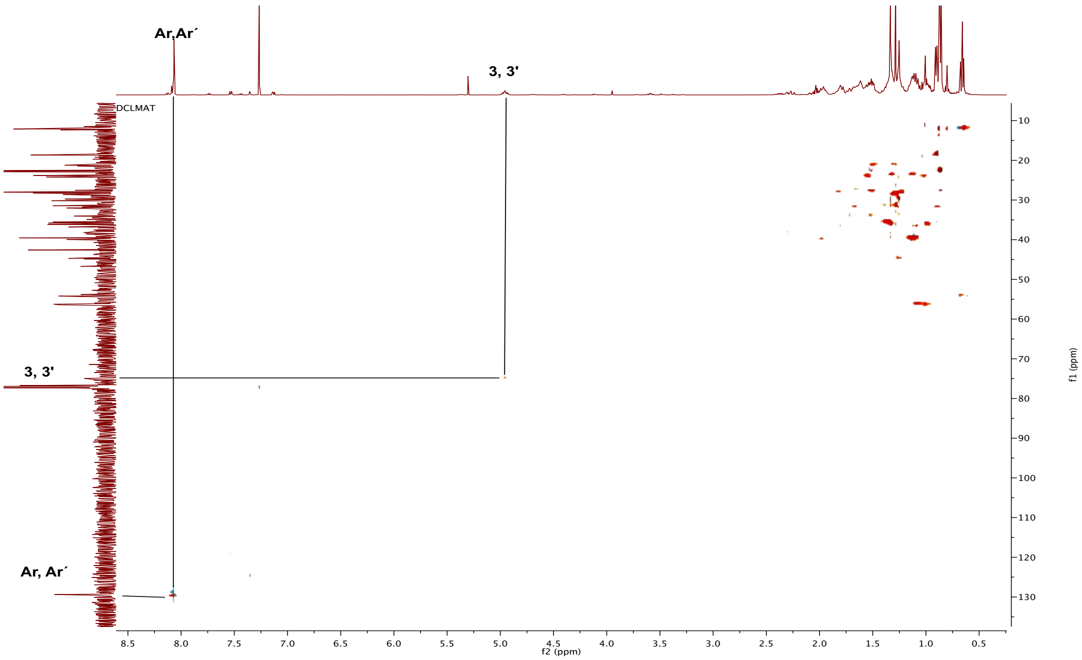

Figure S7. HSQC-NMR spectrum at 500 MHz of Bicholestanol ester (**6**).

Bidiosgenin terephthalate (**8a**).

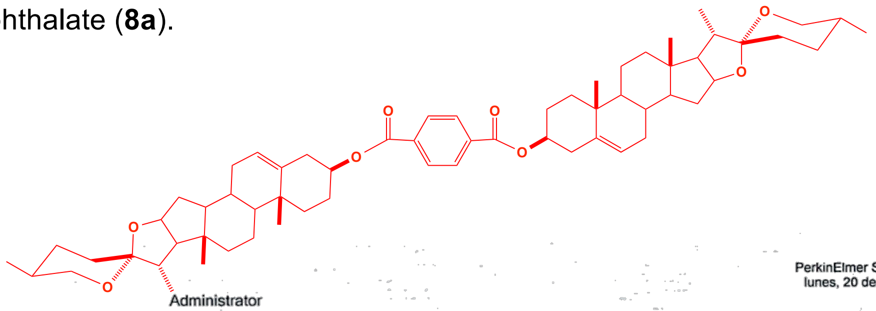

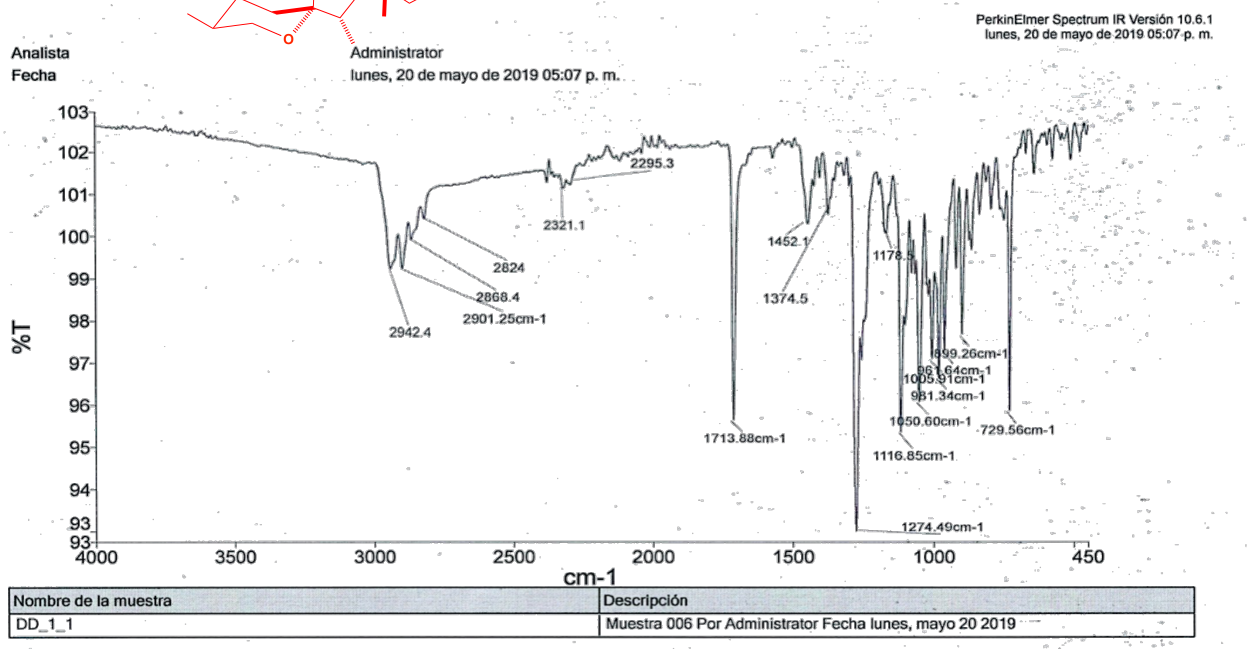

Figure S8. IR spectrum of Bidiosgenin ester (**8a**).

Page: 1

[Elemental Composition]
Data : Dr-Jesus-Sandoval013 Date : 14-Oct-2010 17:31
Sample: STE-2215 LupDD
Note : -
Inlet : Direct Ion Mode : FAB+
RT : 0.36 min Scan#: (1,6)
Elements : C 64/0, H 120/0, O 28/0
Mass Tolerance : 1000ppm, 2mmu if m/z > 2
Unsaturation (U.S.) : -0.5 - 30.0

Observed m/z	Int%				
959.6414	85.7				
Estimated m/z	Error[ppm]	U.S.	C	H	O
959.6401	+1.4	19.5	62	87	8

Figure S9. HRMS data of Bidiosgenin ester (**8a**).

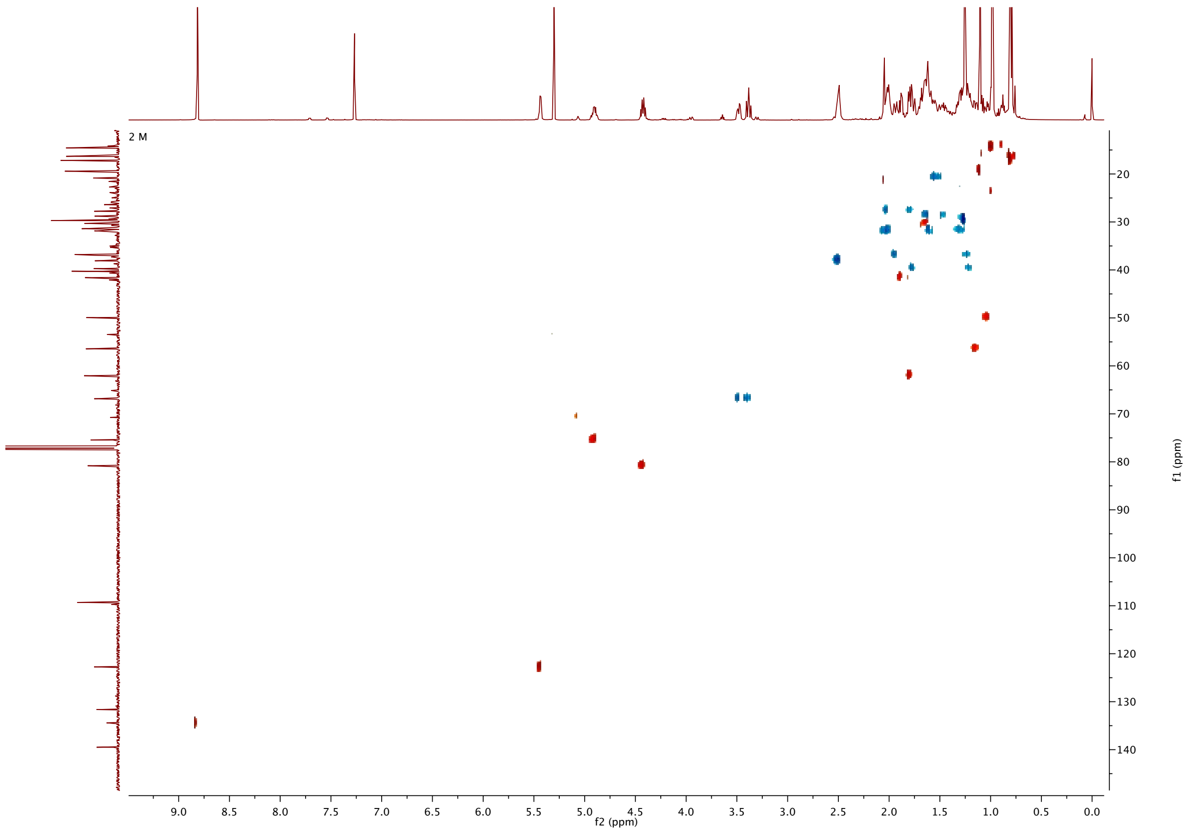

Figure S10. HSQC-NMR spectrum at 600 MHz in $CDCl_3$ of Bidiosgenin ester (**8a**).

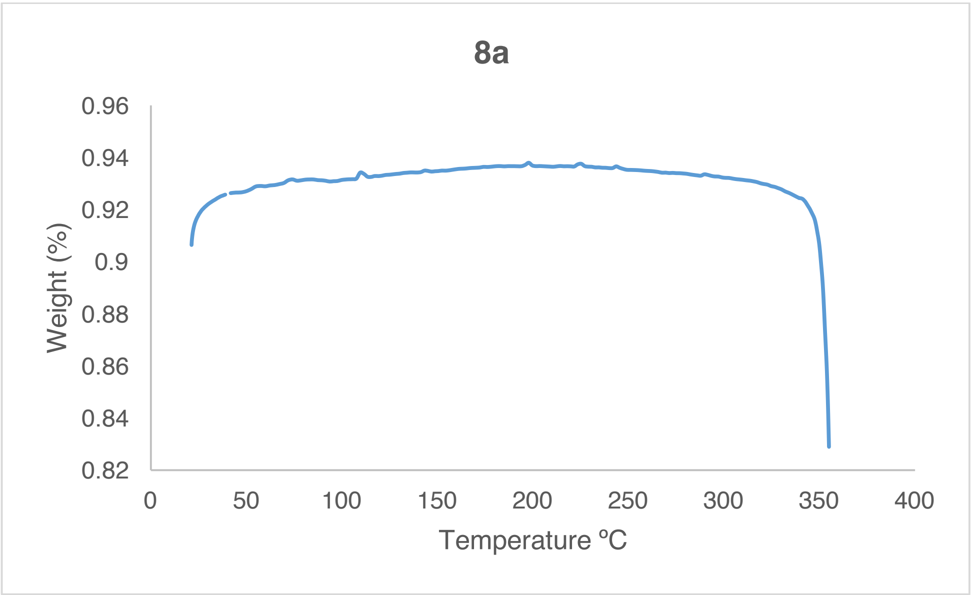

Figure S11. Thermogravimetric analysis of Bidiosgenin ester (**8a**).

Bihecogenin terephthalate (**8b**).

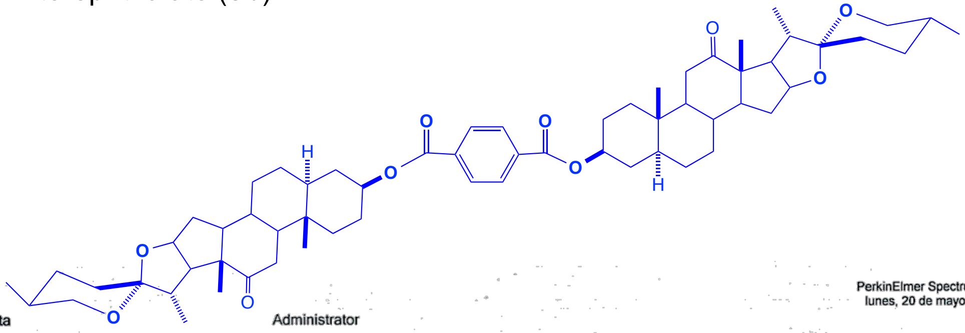

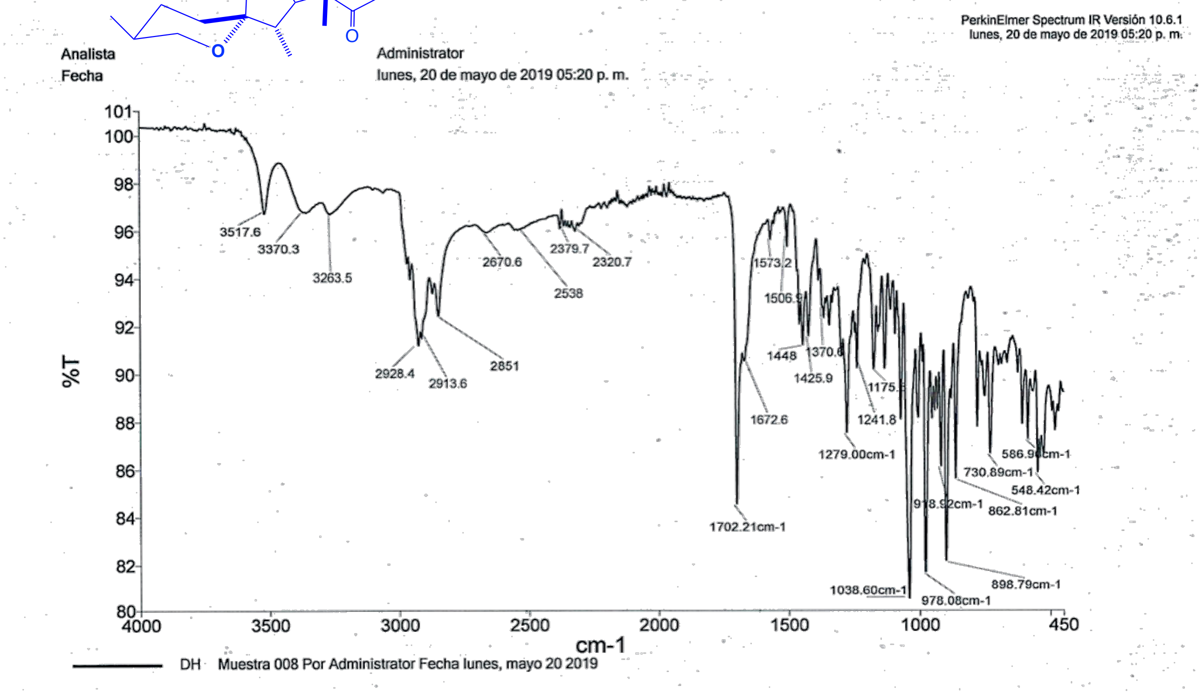

Figure S12. IR spectrum of Bihecogenin ester (**8b**).

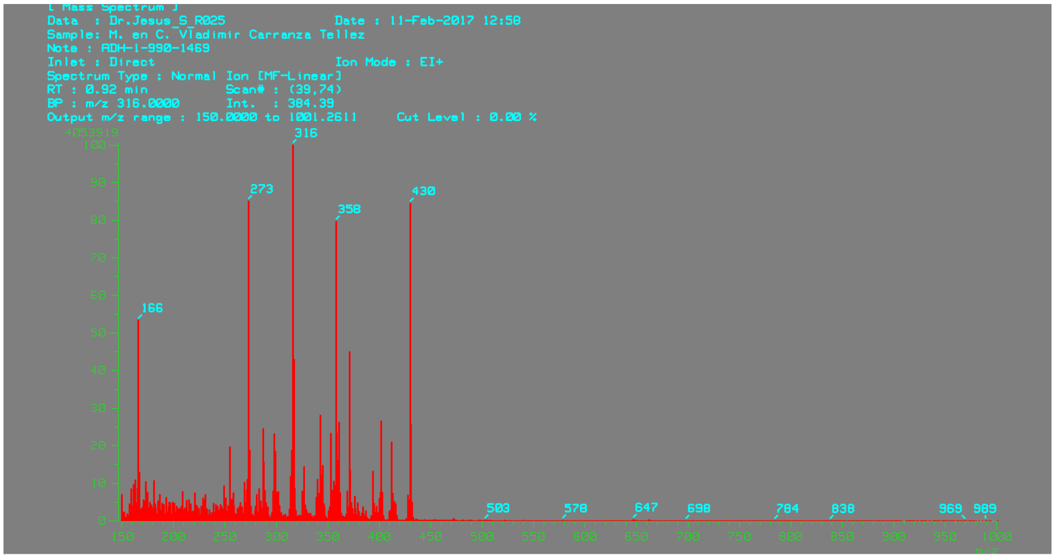

Figure S13. Mass spectrum of Bihecogenin ester (**8b**).

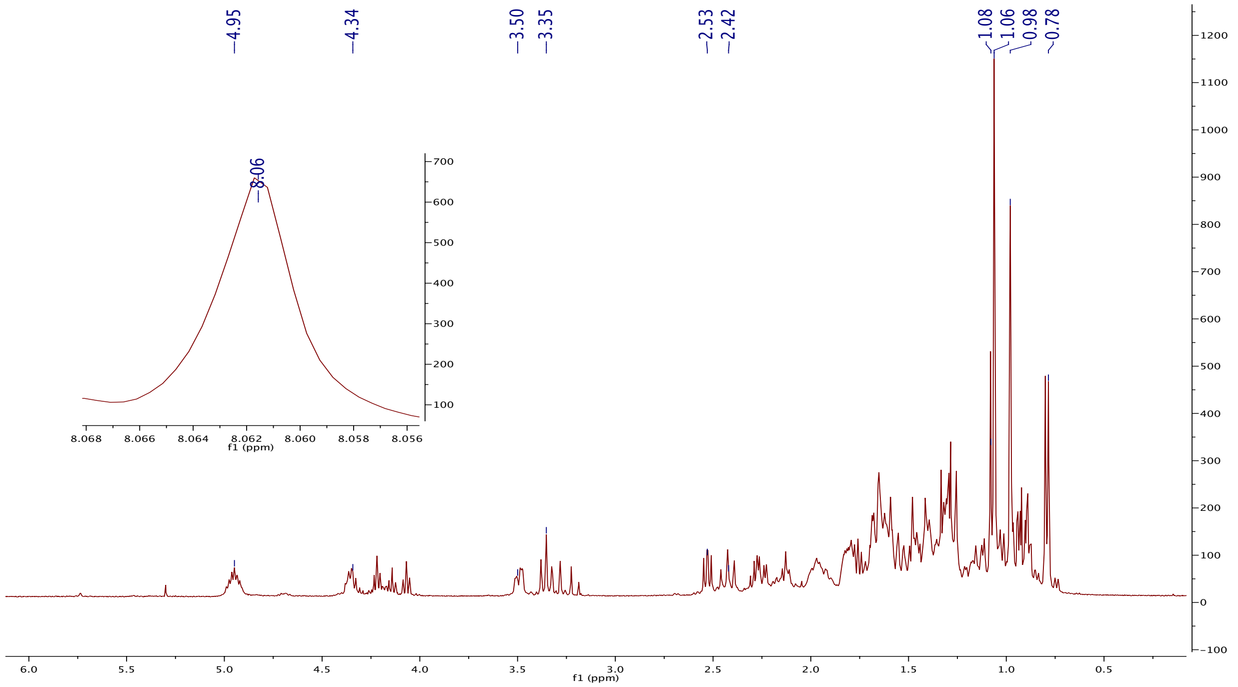

Figure S14. ^{1}H-NMR spectrum at 500 MHz in $CDCl_3$ of Bihecogenin ester (**8b**).

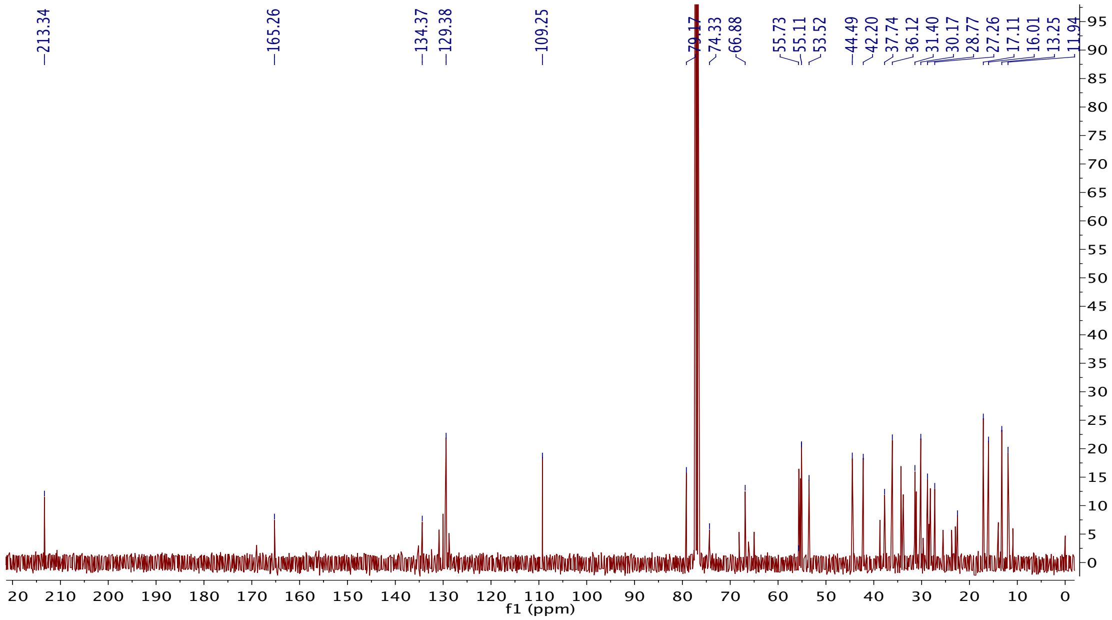

Figure S15. ^{13}C-NMR spectrum at 125 MHz in $CDCl_3$ of Bihecogenin ester (**8b**).

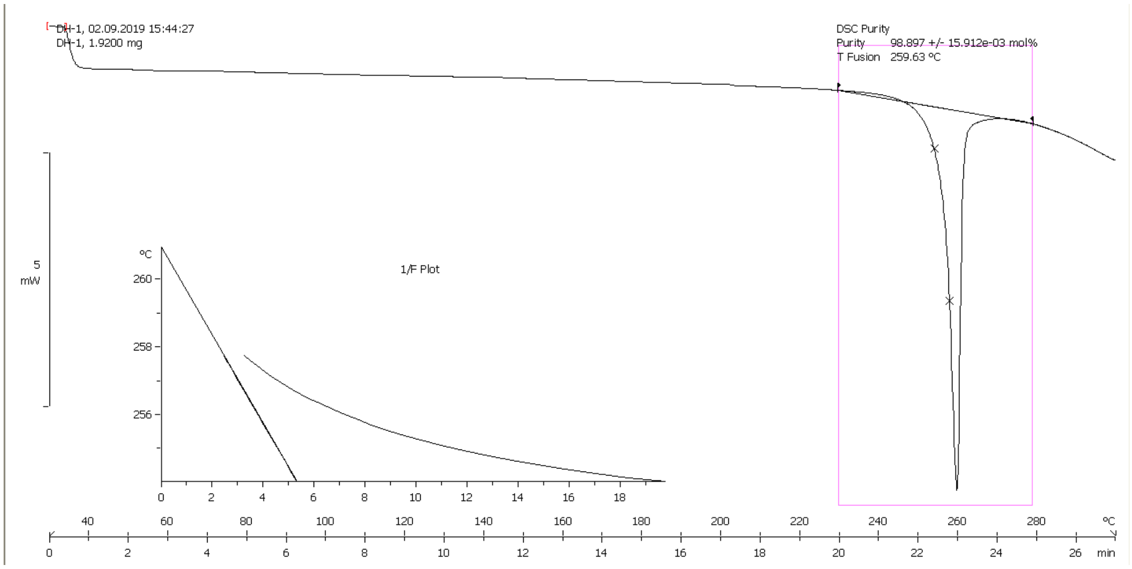

Figure S16. Differential Scanning Calorimetry analysis of Bihecogenin ester (**8b**).

Bisarsasapogenin terephthalate (**8c**)

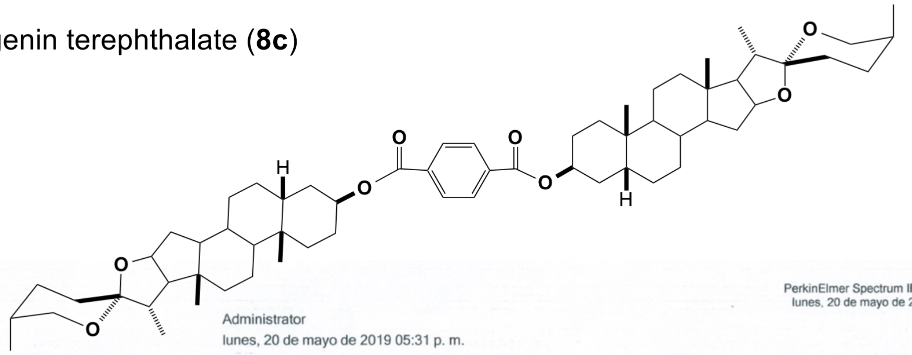

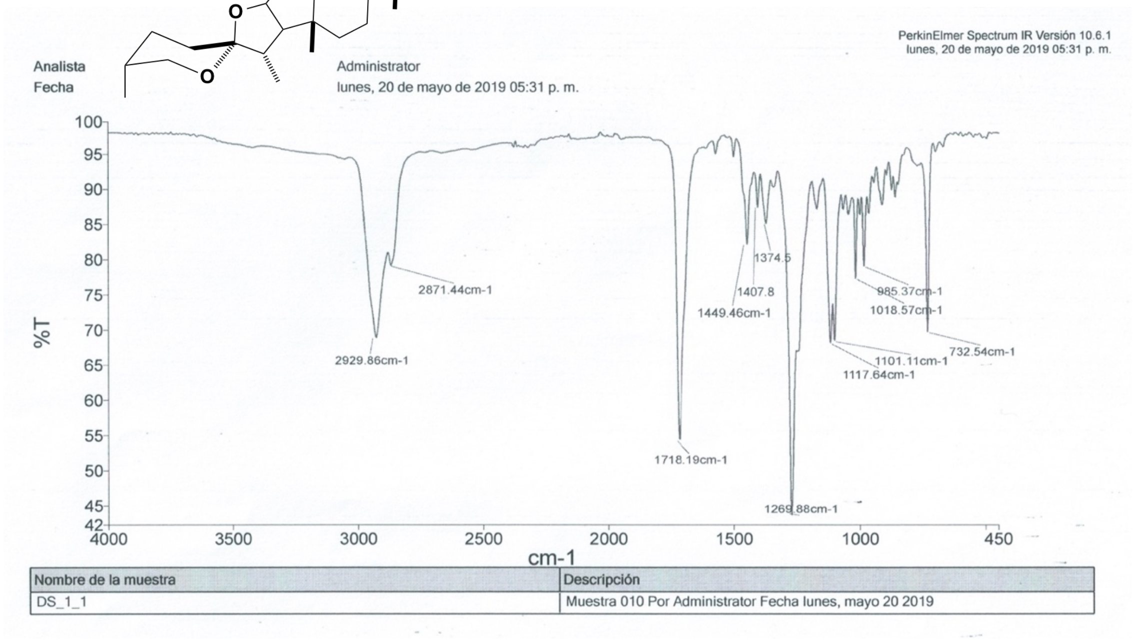

Figure S17. IR spectrum of Bisarsasapogenin ester (**8c**).

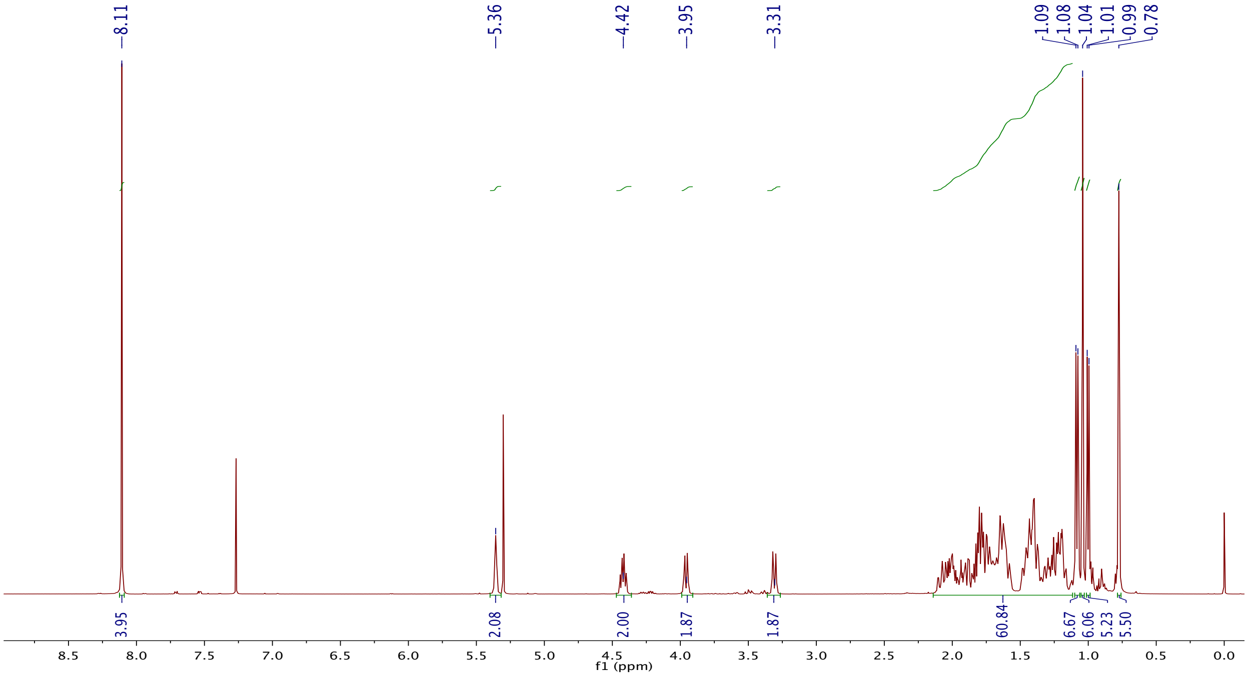

Figure S18. ^{1}H-NMR spectrum at 500 MHz in $CDCl_3$ of Bisarsasapogenin ester (**8c**).

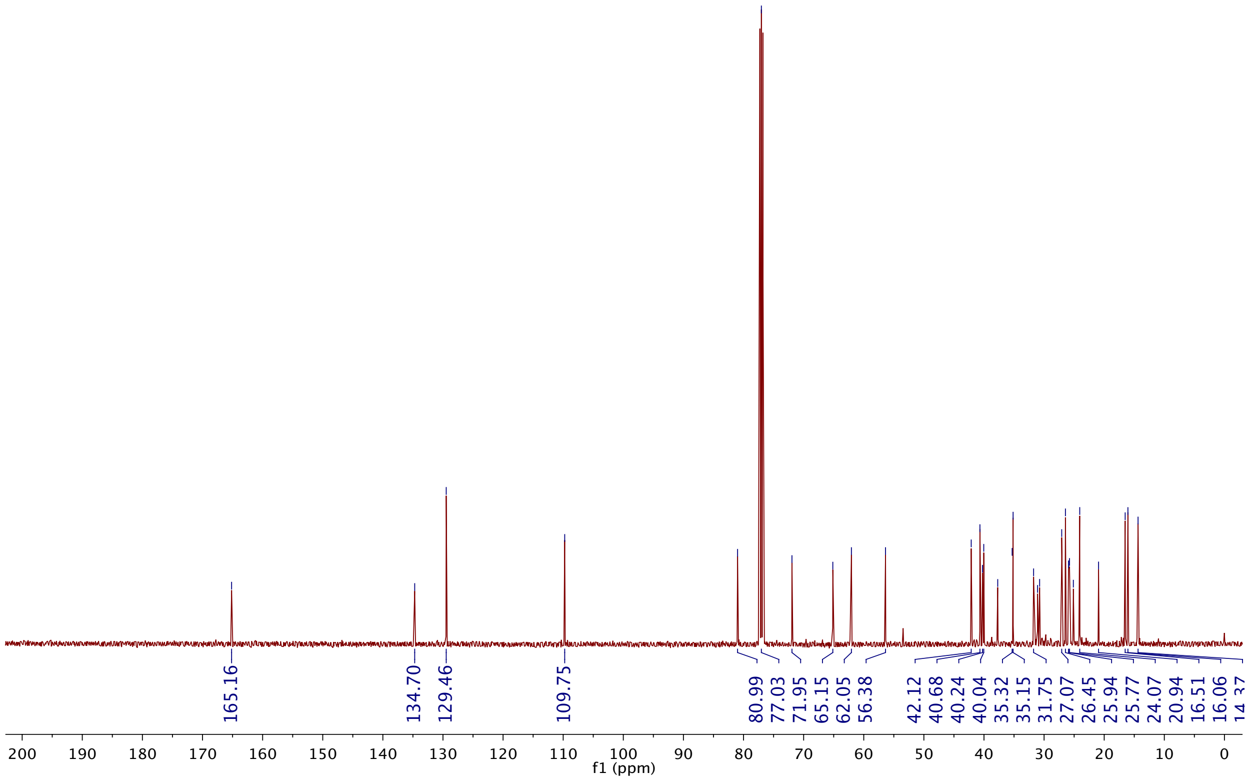

Figure S19. ^{13}C-NMR spectrum at 125 MHz in $CDCl_3$ of Bisarsasapogenin ester (**8c**).

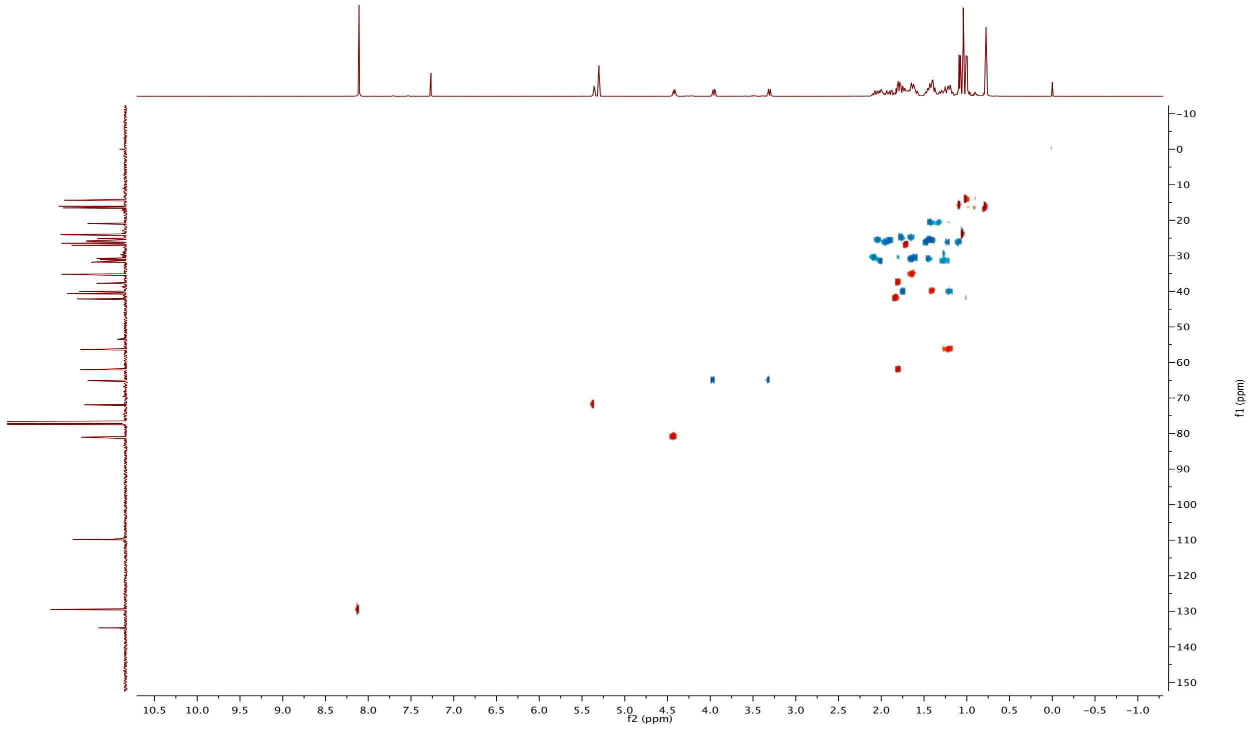

Figure S20. HSQC-NMR spectrum at 500 MHz in $CDCl_3$ of Bisarsasapogenin ester (**8c**).

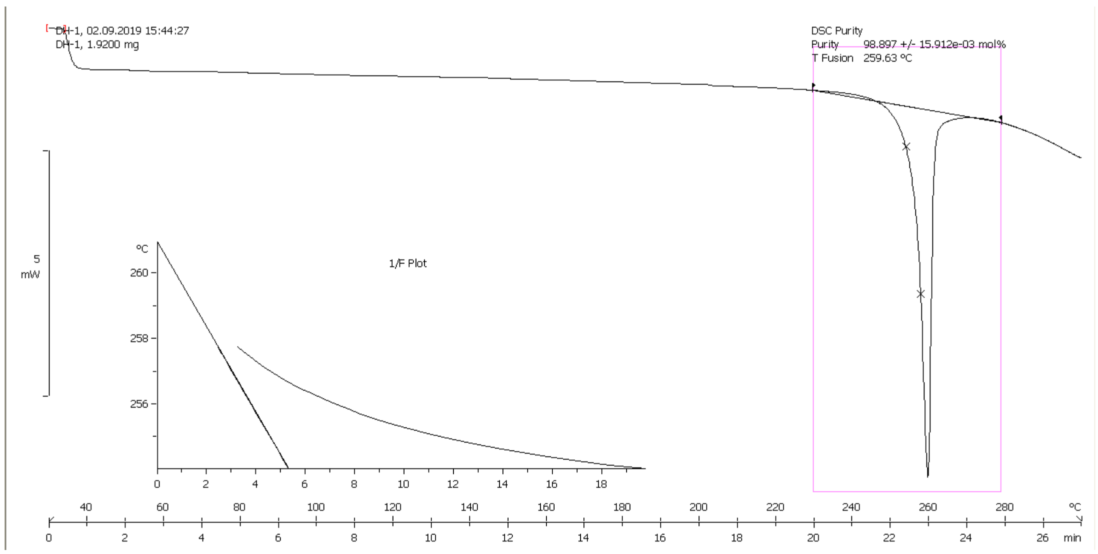

Figure S21. Differential Scanning Calorimetry analysis of Bisarsasapogenin ester (**8c**).

Bi-23-acetyldiosgenin terephthalate (**10a**).

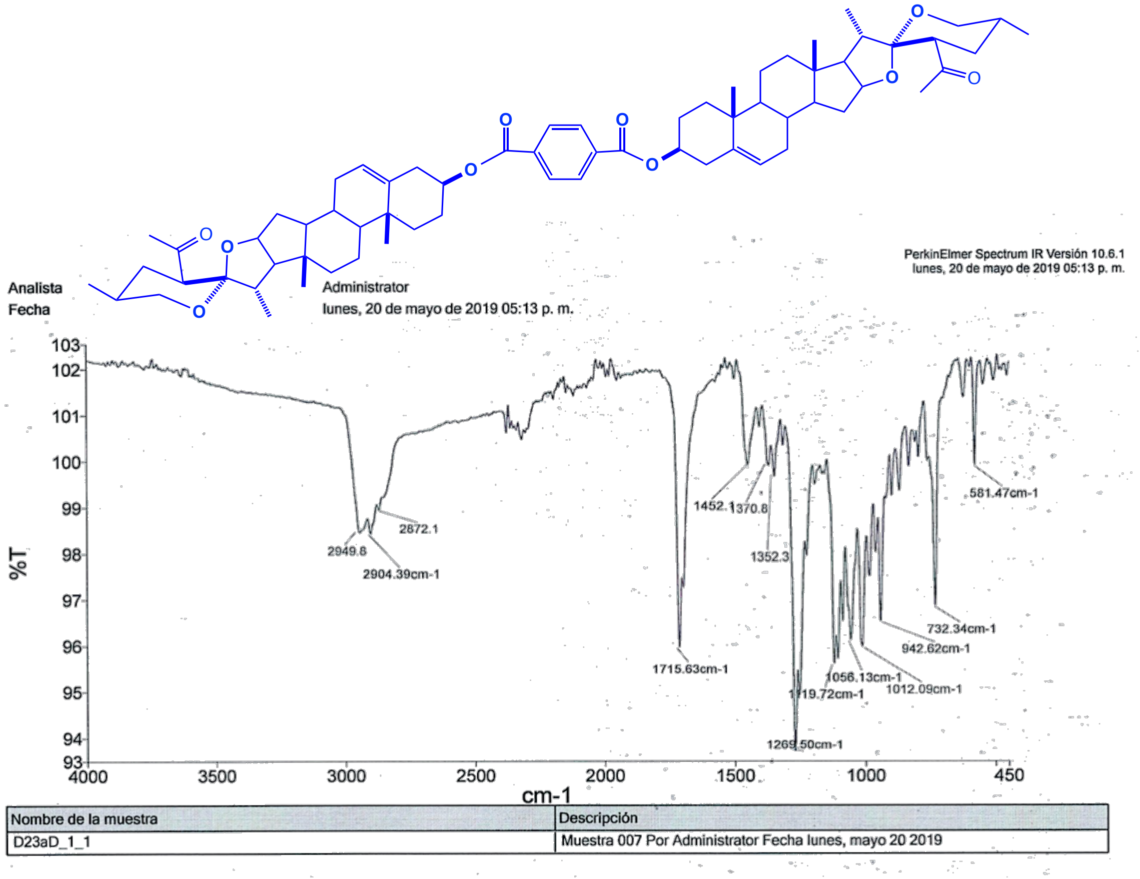

Figure S22. IR spectrum of Bi-23-acetyldiosgenin ester (**10a**).

```
[ Elemental Composition ]                                              Page: 1
Data   : Dr-Jesus-Sandoval039             Date : 08-Oct-2010 13:20
Sample: 65-STE-2216     Lup-23aD
Note : Luis-Velasco
Inlet : Direct                            Ion Mode : FAB+
RT : 9.15 min                             Scan#: (65,76)
Elements : C 70/1, H 95/1, O 12/1
Mass Tolerance      : 1000ppm, 2mmu if m/z > 2
Unsaturation (U.S.) : 0.0 - 50.0

Observed m/z   Int%
 1043.6609      41.2
Estimated m/z  Error[ppm]   U.S.     C     H     O
 1043.6612       -0.3       21.5    66    91    10
```

Figure S23. Mass spectrum data of Bi-23-acetyldiosgenin ester (**10a**).

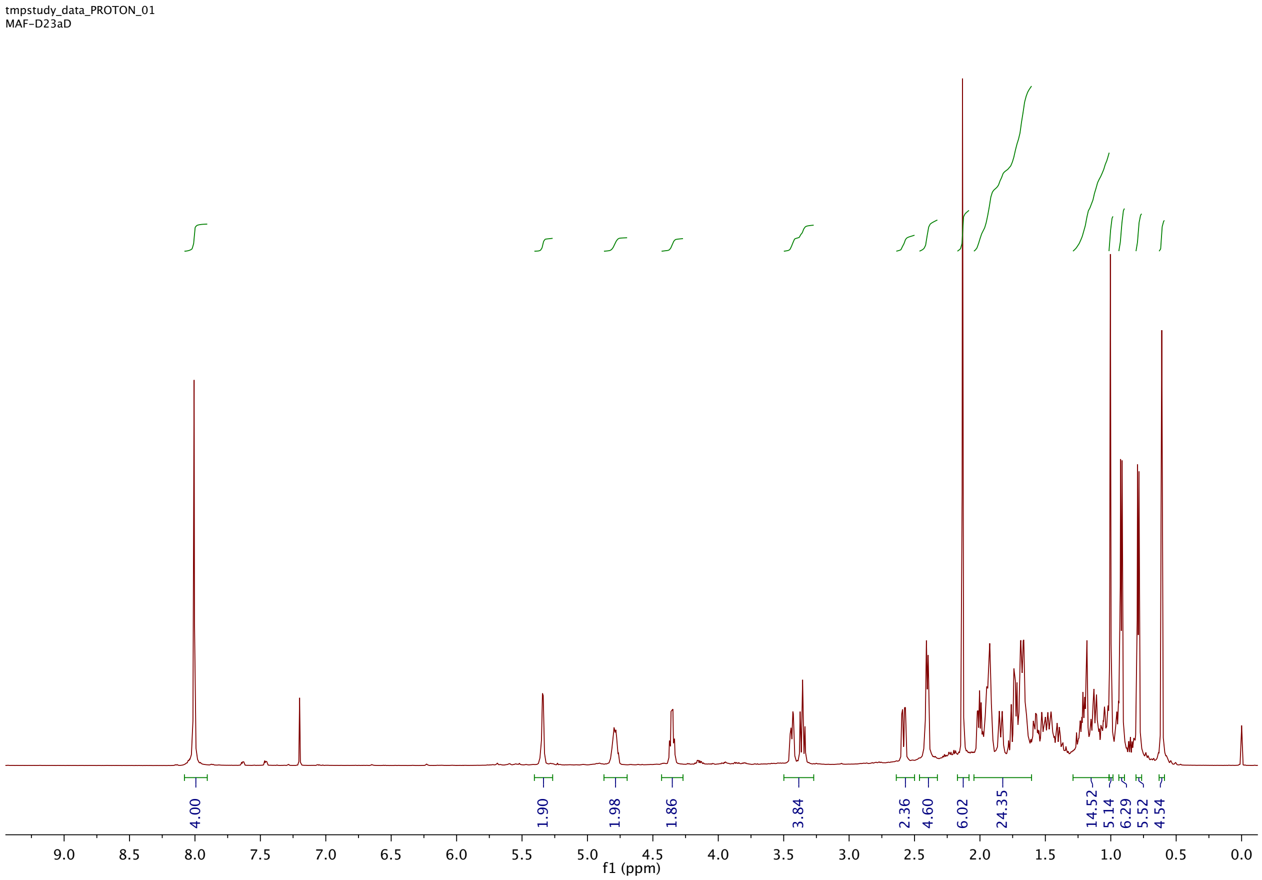

Figure S24. ^{1}H-NMR spectrum at 600 MHz in $CDCl_3$ of Bi-23-acetyldiosgenin ester (**10a**).

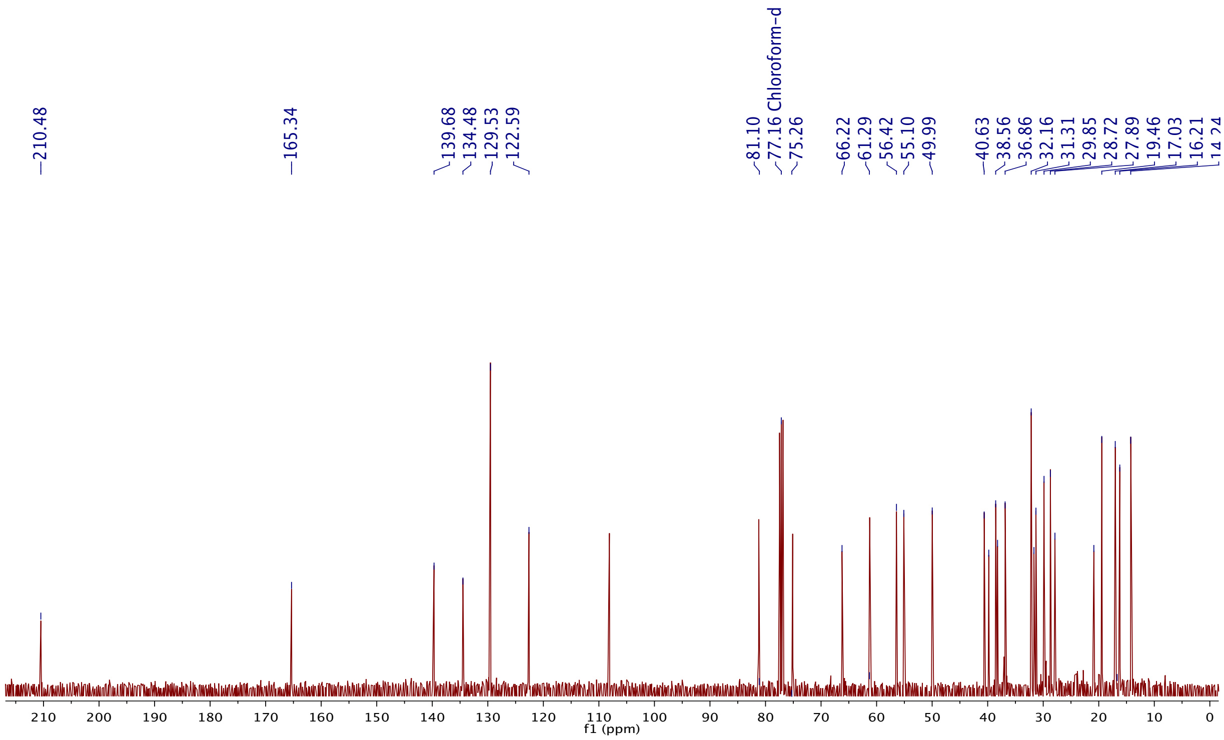

Figure S25. ^{13}C-NMR spectrum at 150 MHz in $CDCl_3$ of Bi-23-acetyldiosgenin ester (**10a**).

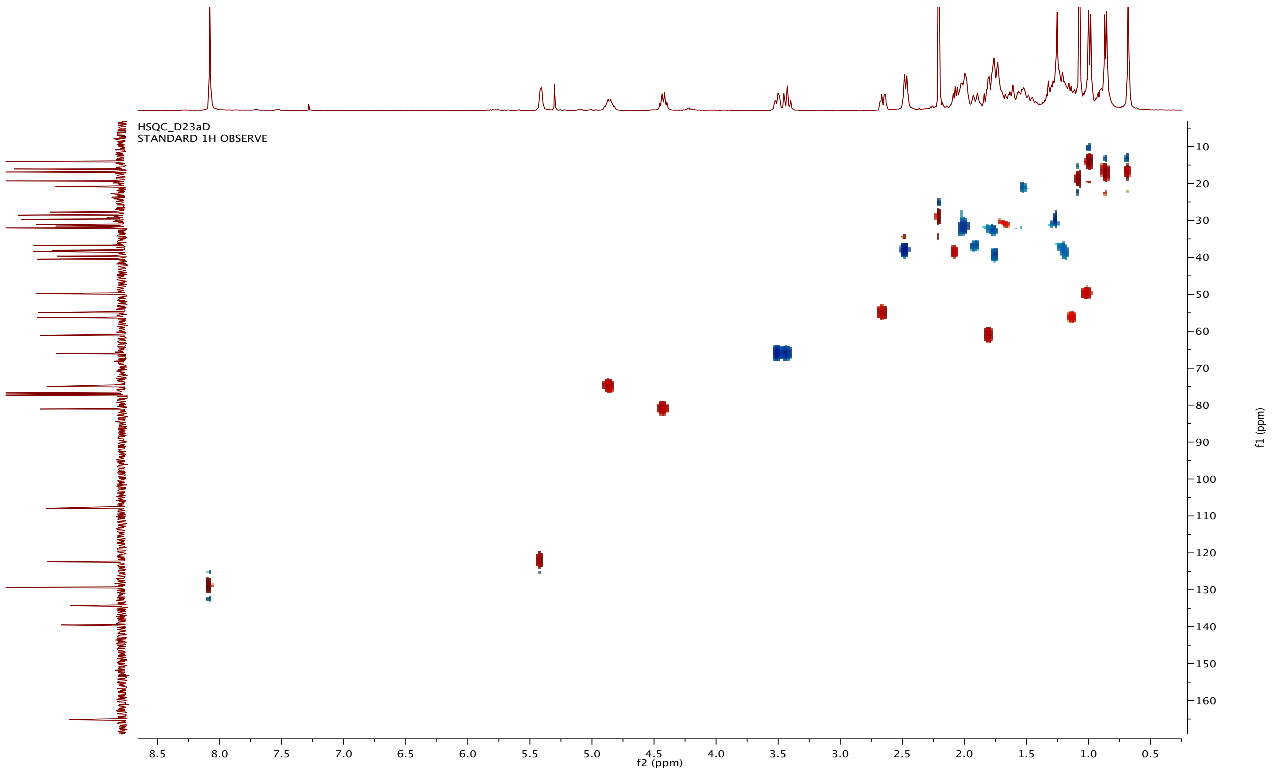

Figure S26. HSQC-NMR spectrum at 500 MHz in $CDCl_3$ of Bi-23-acetyldiosgenin ester (**10a**).

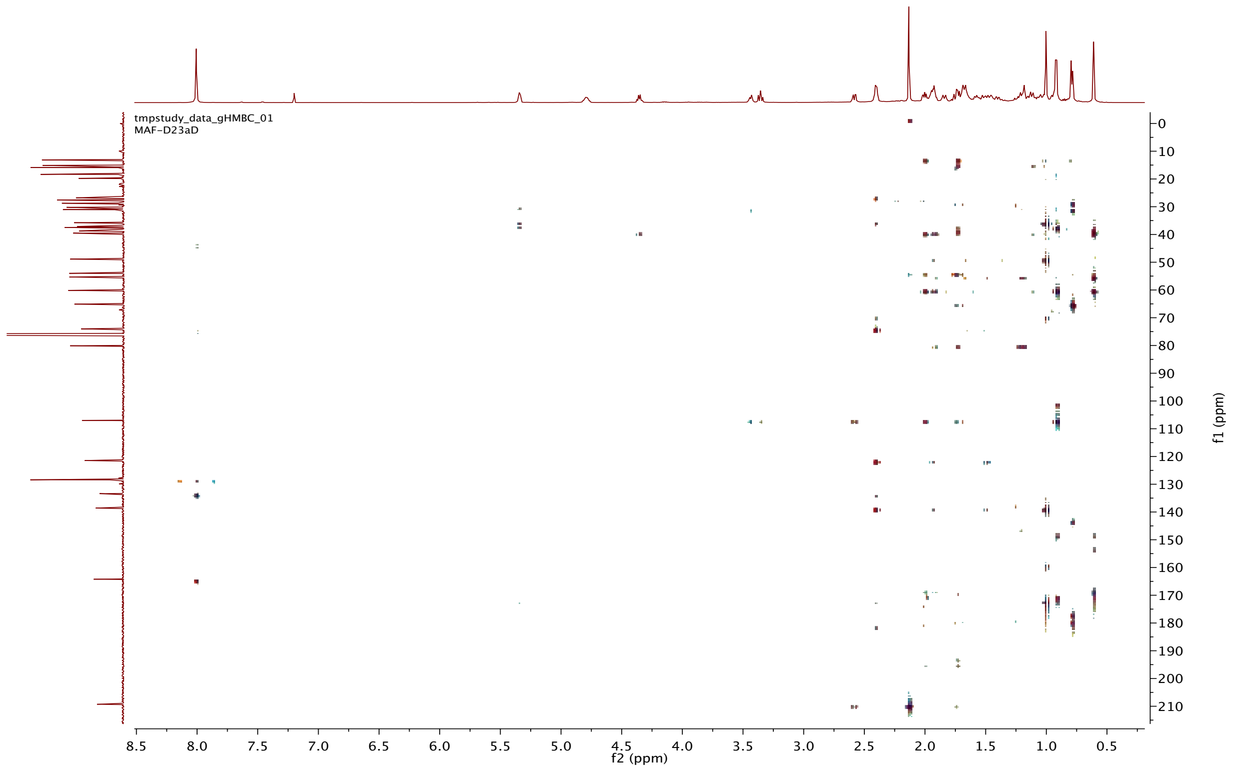

Figure S27. HMBC-NMR experiment al 600 MHz in $CDCl_3$ of Bi-23-acetyldiosgenin ester (**10a**).

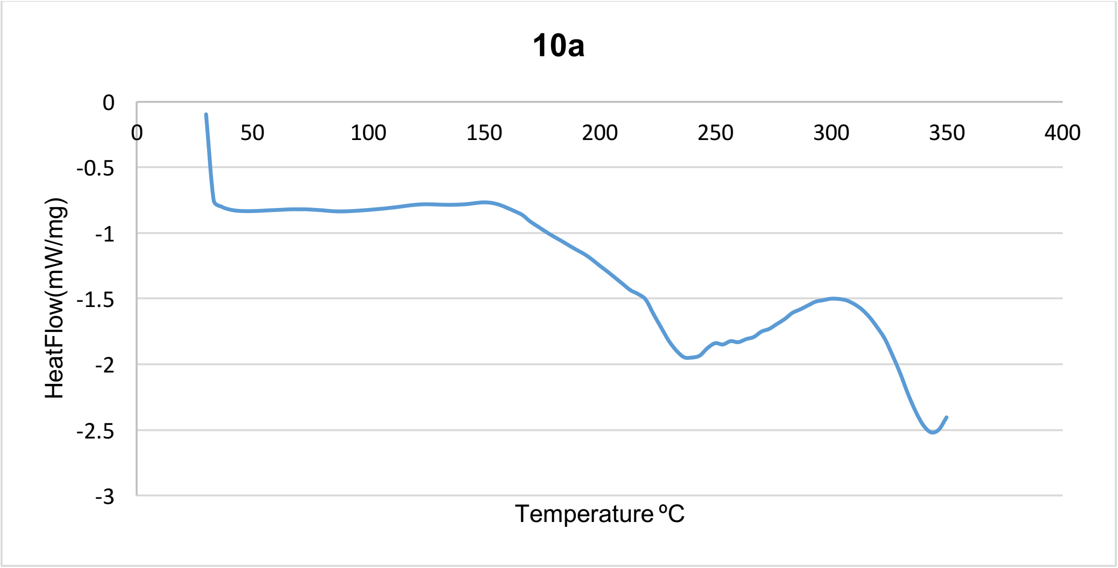

Figure S28. Differential Scanning Calorimetry analysis of Bi-23-acetyldiosgenin ester (**10a**).

.

Bi-23-acetylhecogenin terephthalate (**10b**).

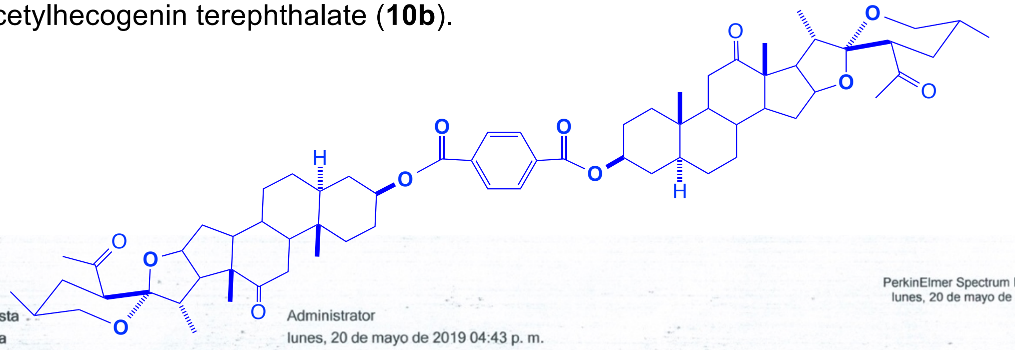

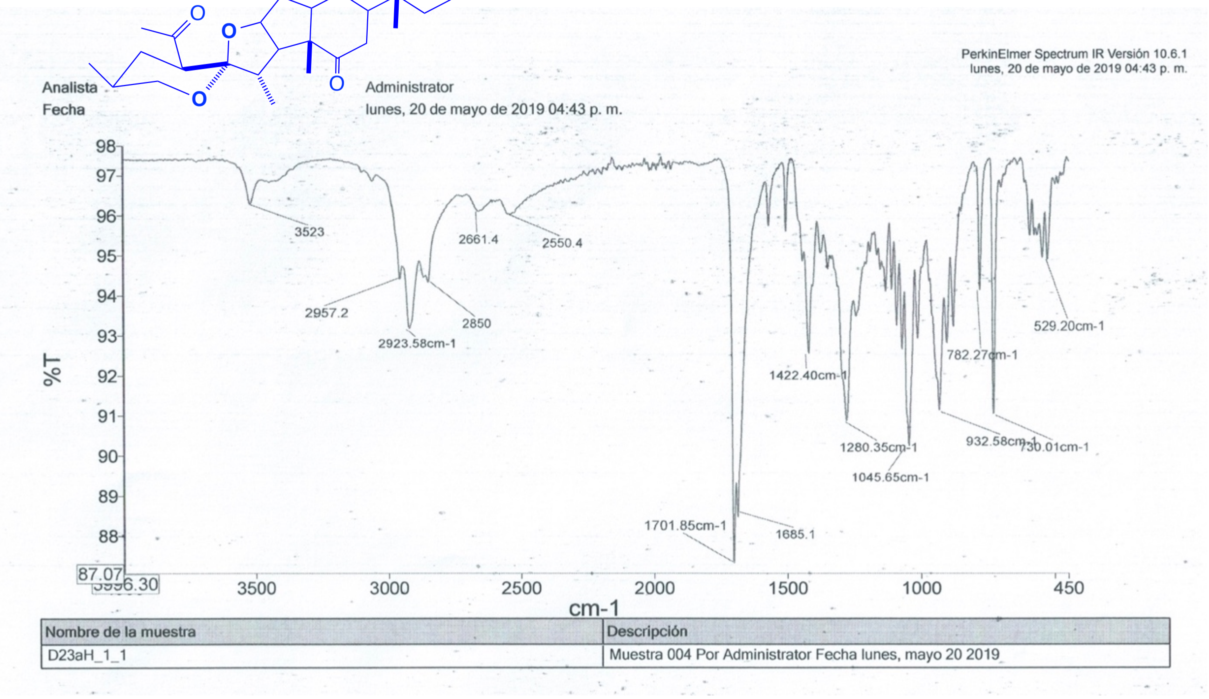

Figure S29. IR spectrum of Bi-23-acetylhecogenin ester (**10b**).

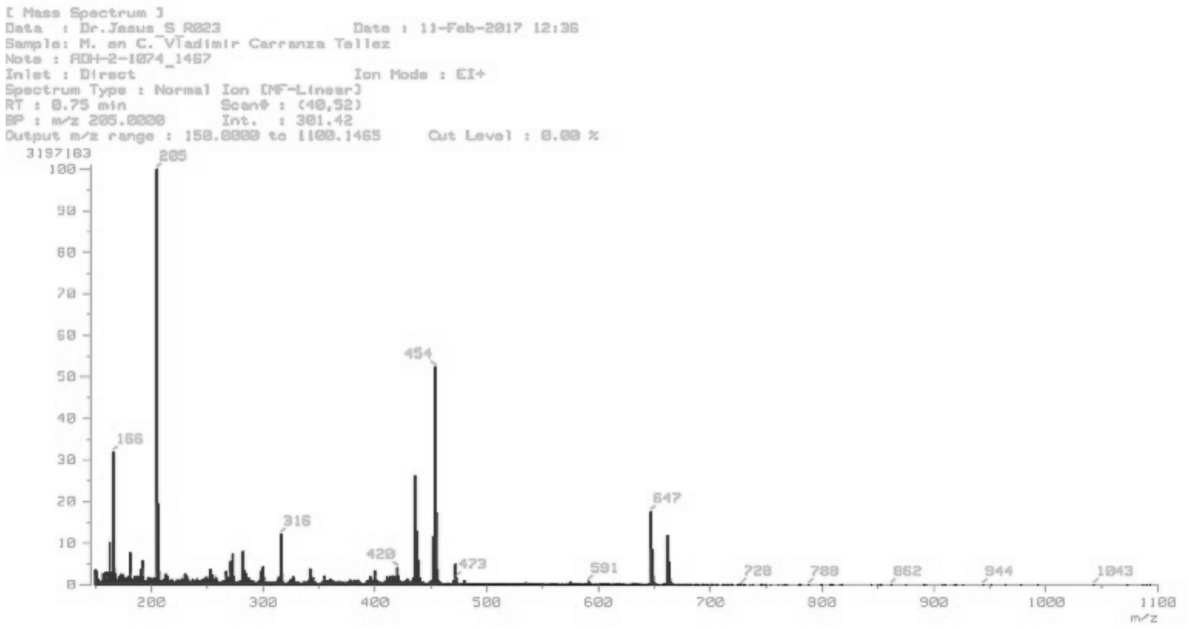

Figure S30. Mass spectrum of Bi-23-acetylhecogenin ester (**10b**).

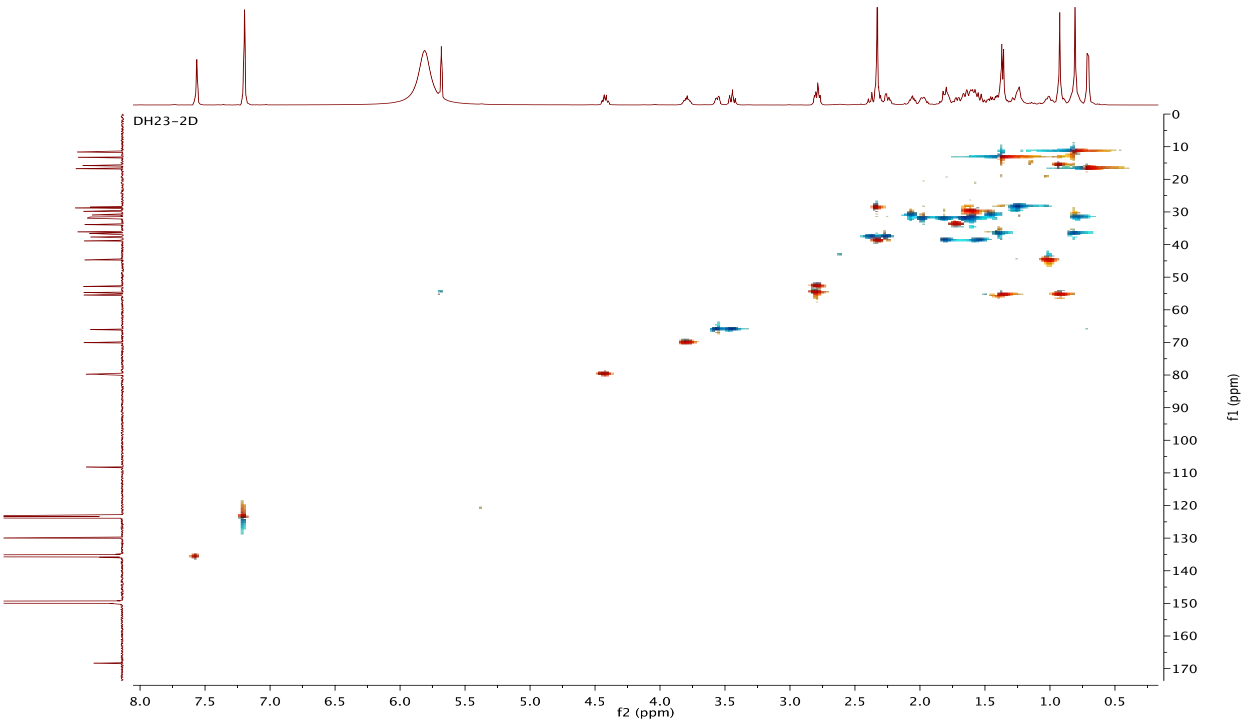

Figure S31. HSQC-NMR spectrum at 500 MHz in $CDCl_3$ of Bi-23-acetylhecogenin ester (**10b**).

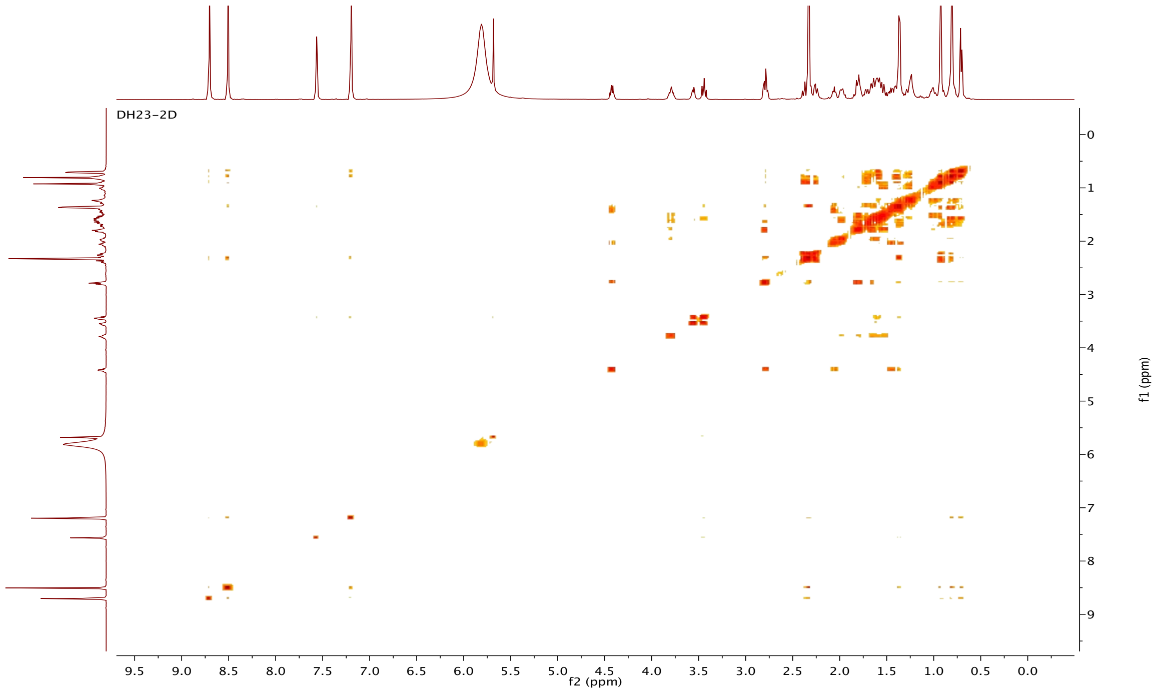

Figure S32. COSY-NMR spectrum at 500 MHz in $CDCl_3$ of Bi-23-acetylhecogenin ester (**10b**).

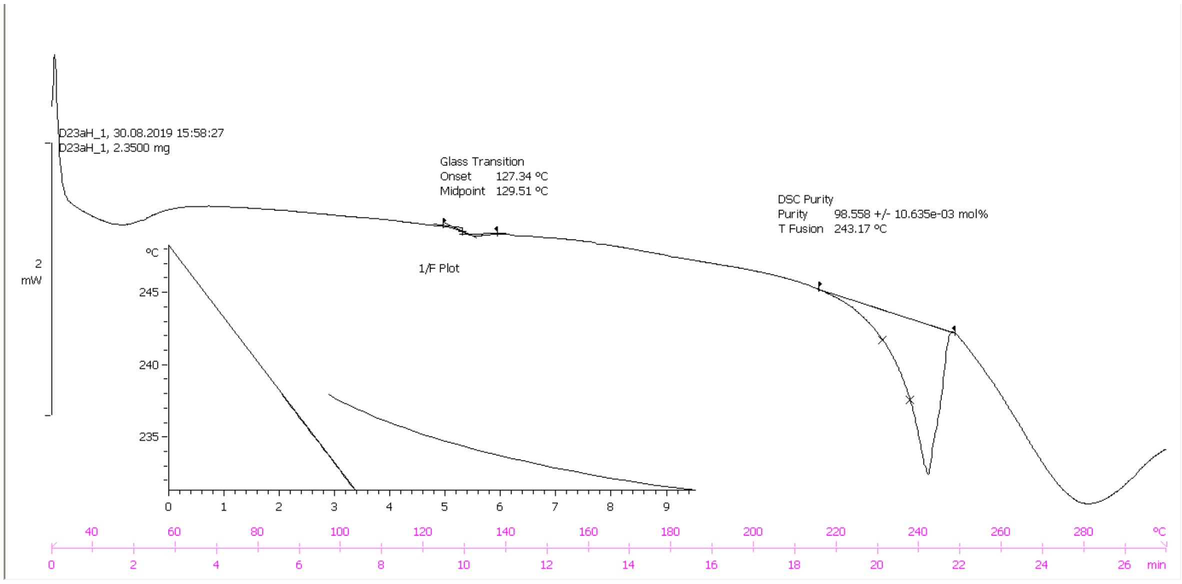

Figure S33. Differential Scanning Calorimetry analysis of Bi-23-acetylhecogenin ester (**10b**).

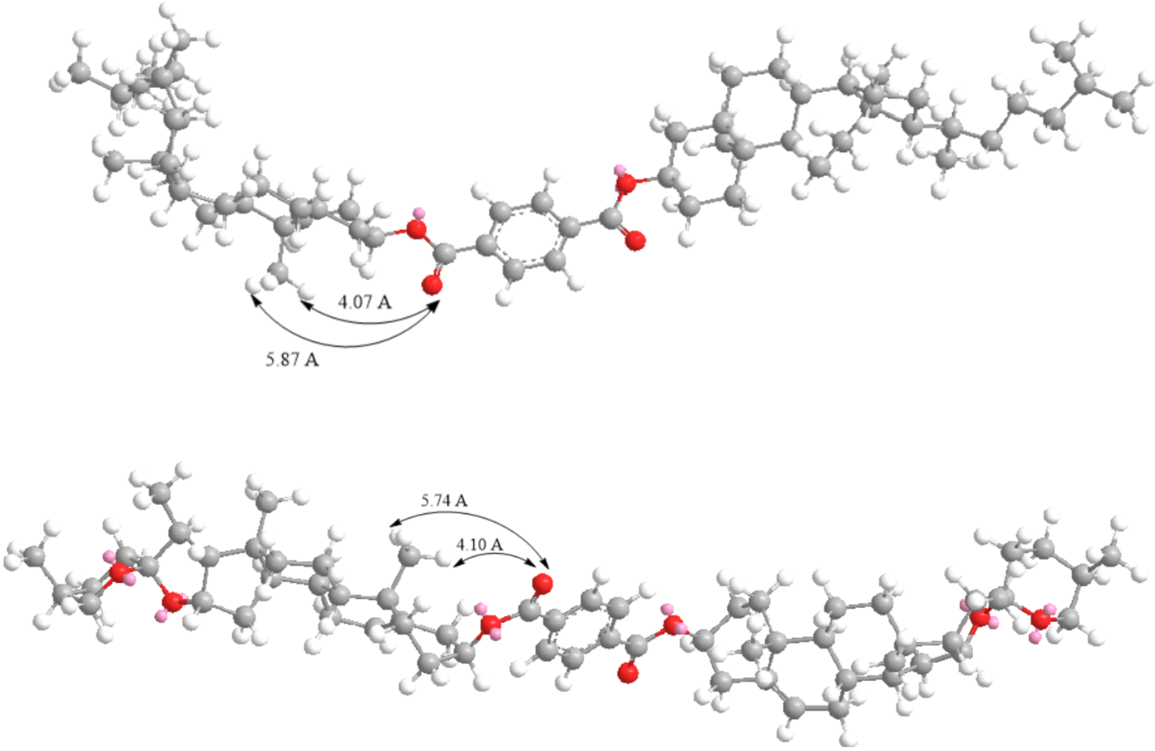

Figure S34. Molecular structure with MM2 energy minimization method for series: 5α (**6**, top) and 5β (**8c**, down).

Scanning Electron Microscopy (SEM) Images.

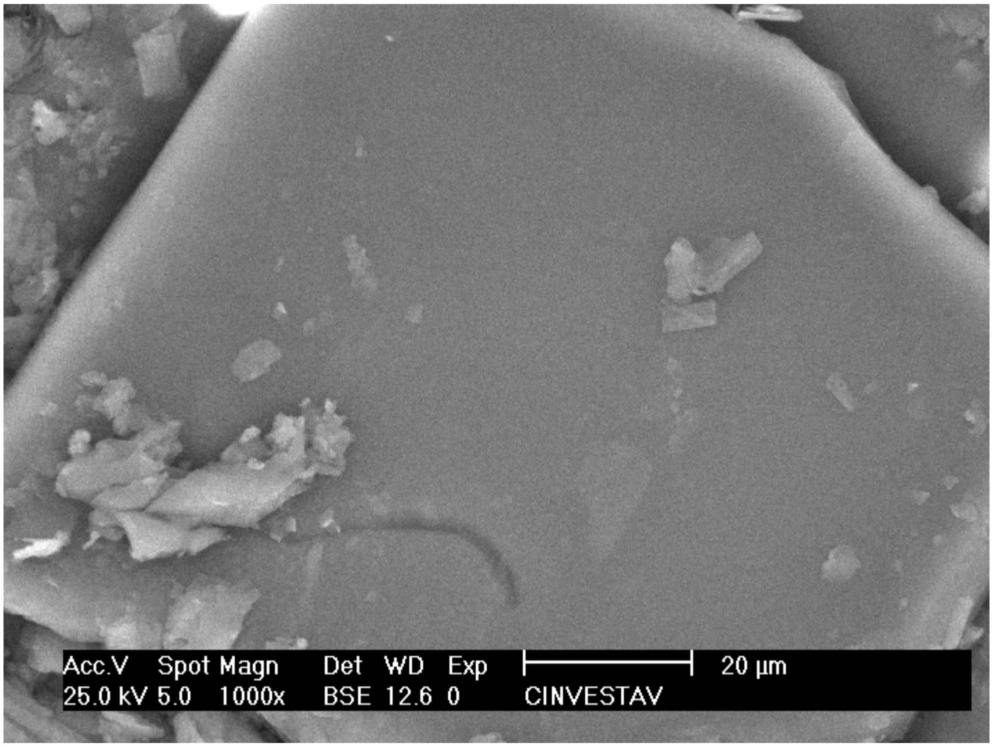

Figure S35 Bicholesterol ester (**5**) in hexane/ EtOAc showing membrane-shaped structures.

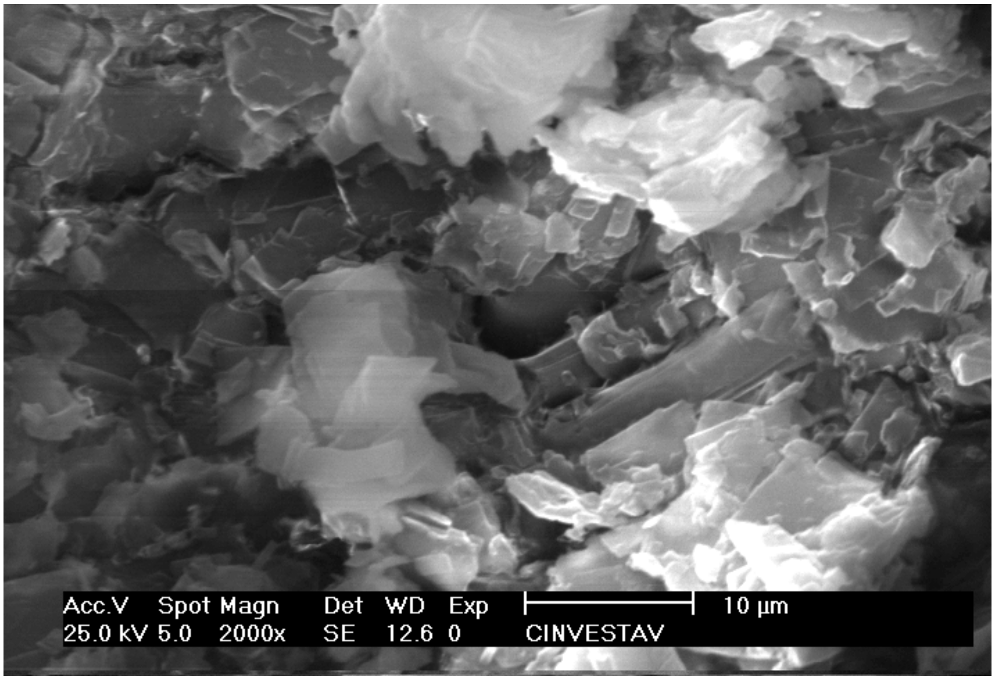

Figure S36 Bicholesterol ester (**5**) in hexane/ EtOAc showing membrane-shaped structures.

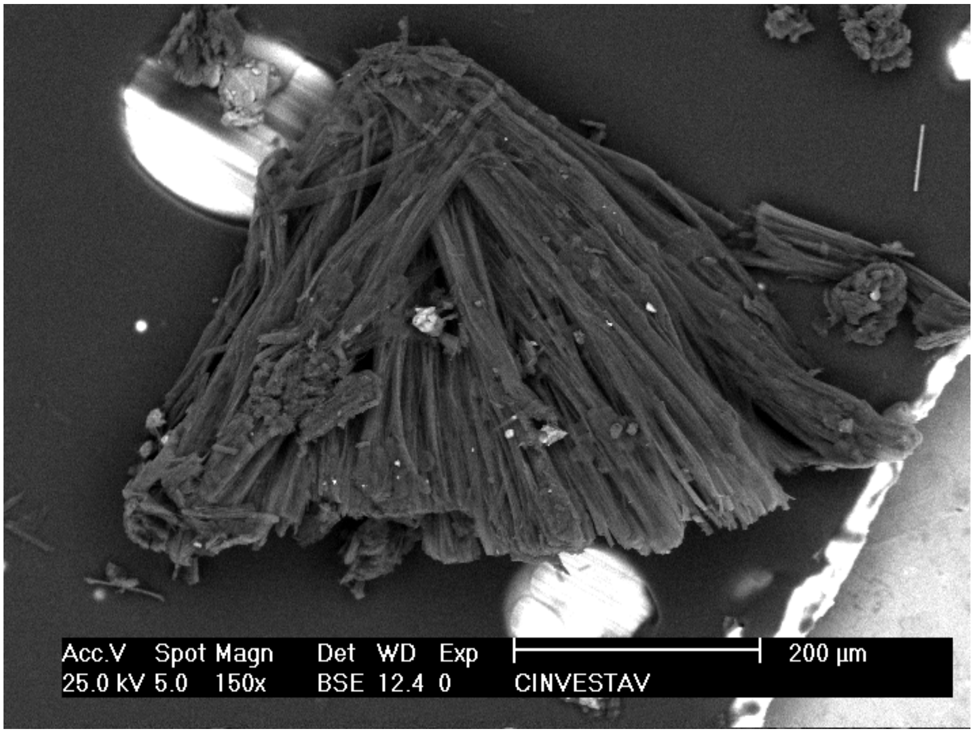

Figure S37. Bicholesterol ester (**5**) EtOAc showing strand-shaped structures.

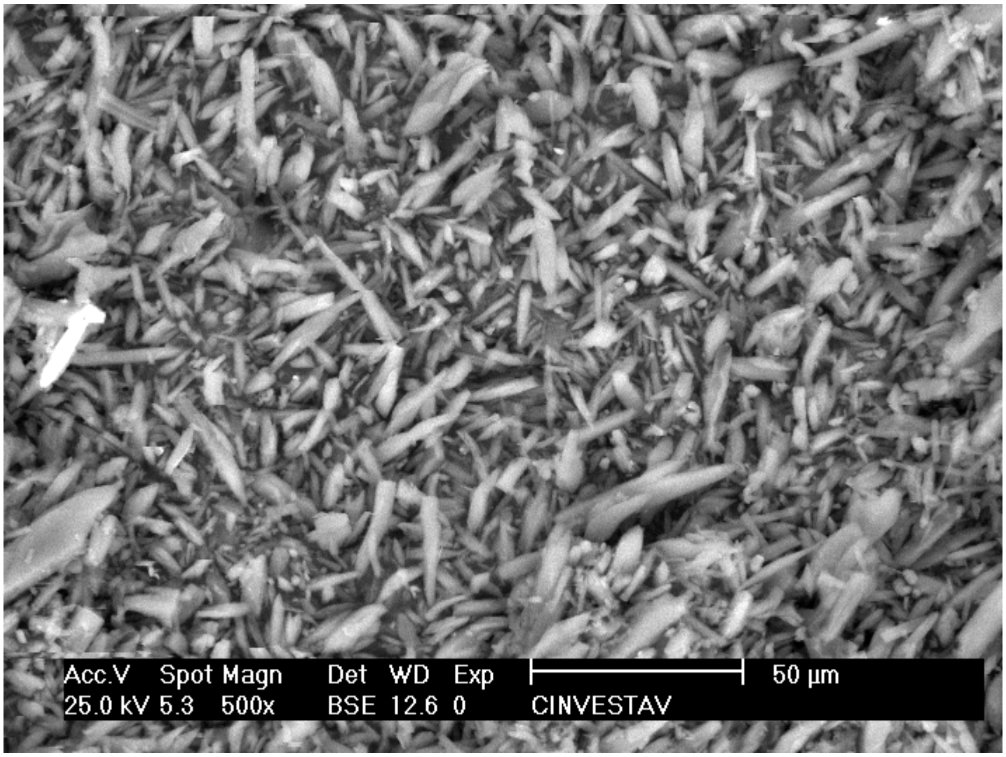

Figure S38. Bidiosgenin ester (**8a**) in EtOAc showing strand-shaped structures.

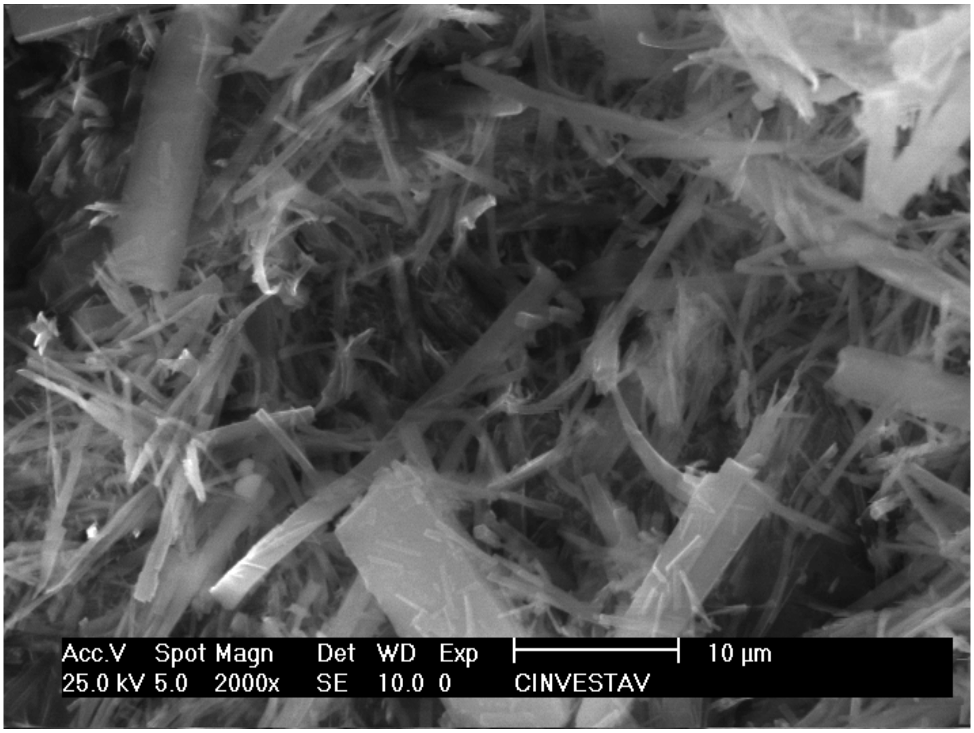

Figure S39. Bidiosgenin ester (**8a**) in $CHCl_3$/MeOH.

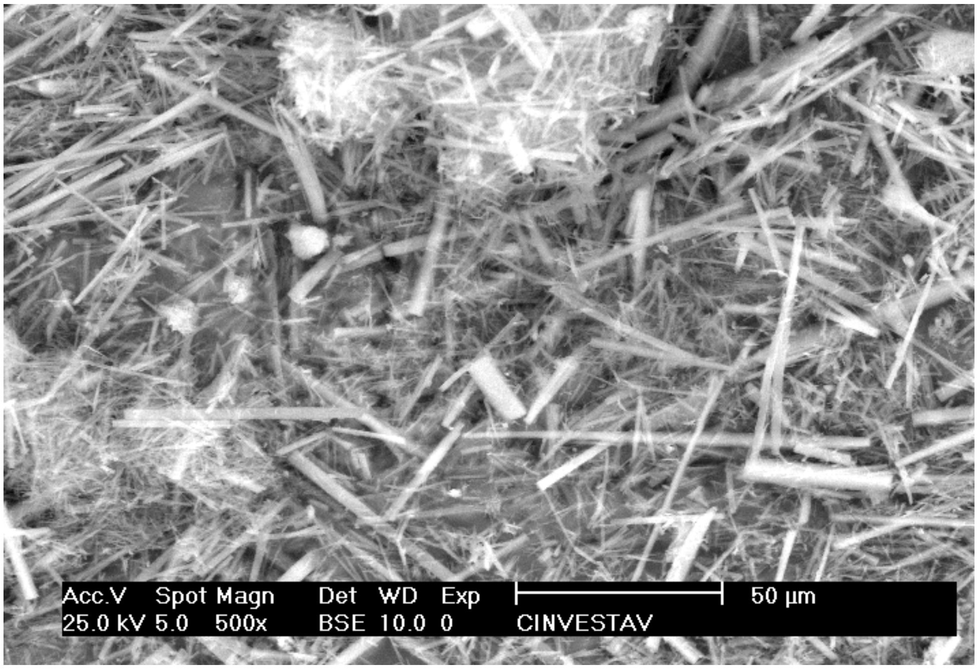

Figure S40. Bidiosgenin ester (**8a**) in $CHCl_3$/MeOH

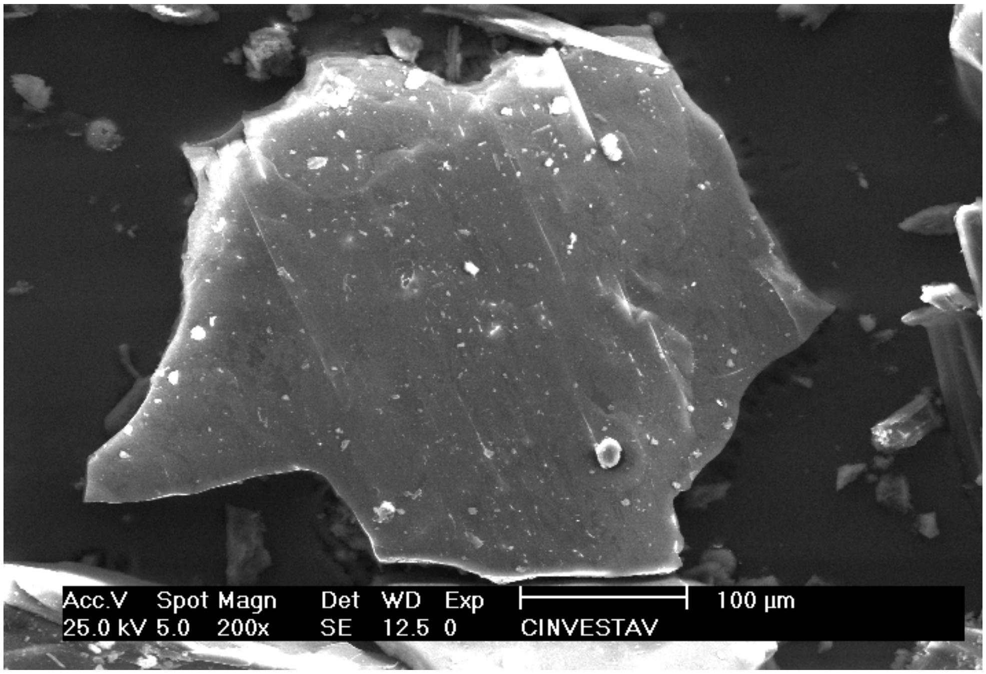

Figure S41. Bisarsasapogenin ester (**8c**) in hexane/EtOAc showing membrane-shaped structures.

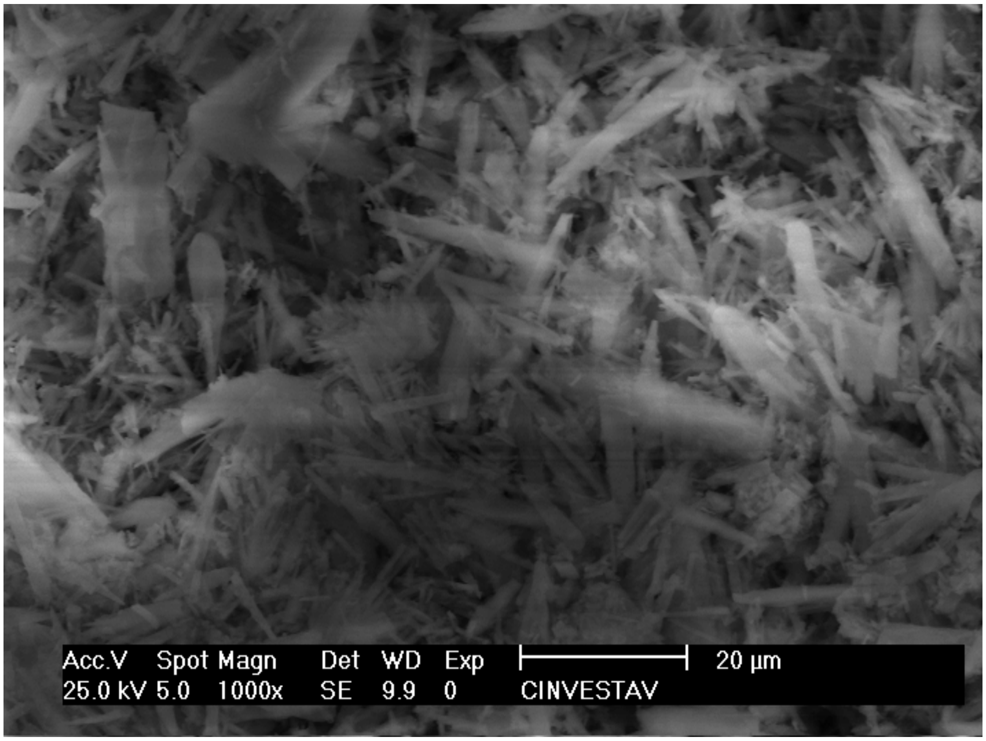

Figure S42. Bi-23-acetyldiosgenin ester (**10a**) in EtOAc showing strand-shaped structures.

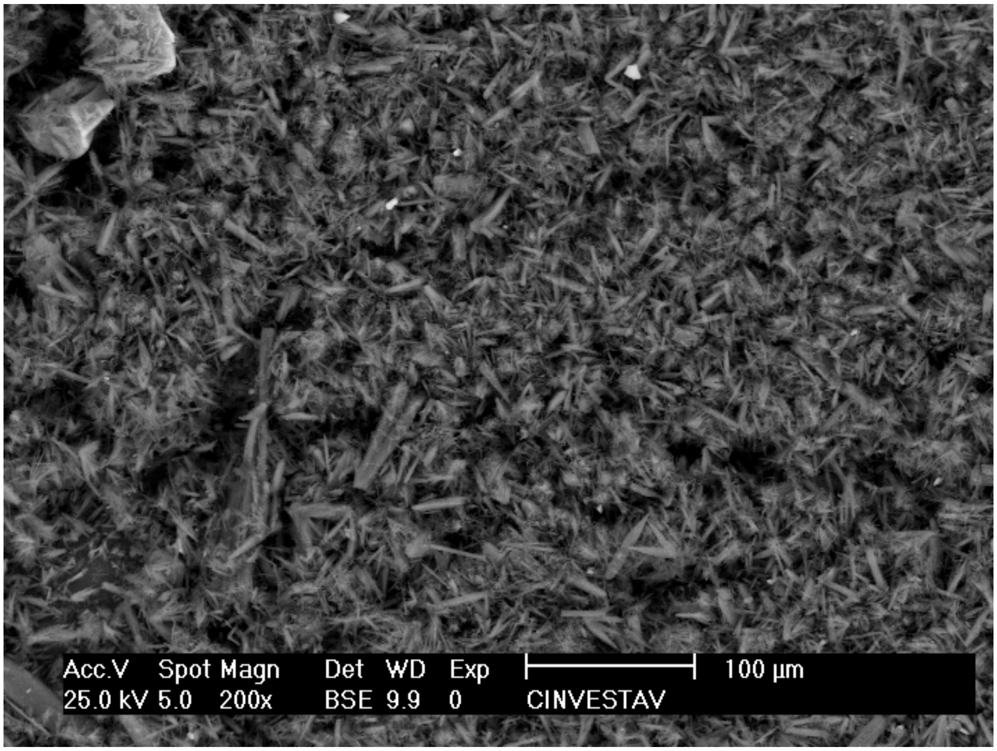

Figure S43. Bi-23-acetyldiosgenin ester (**10a**) in EtOAc showing strand-shaped structures.

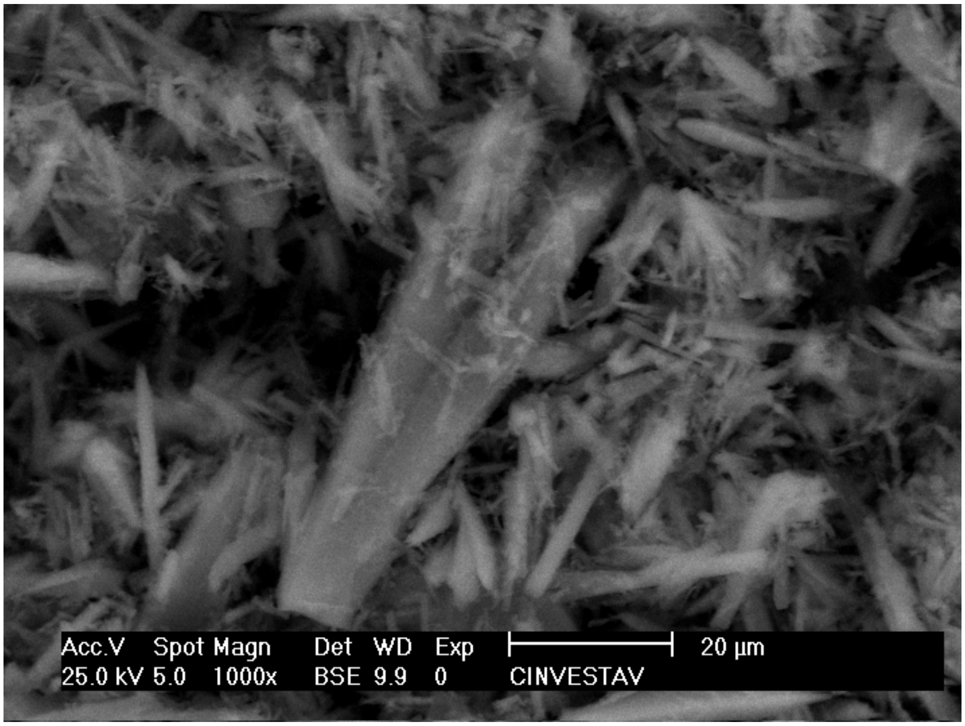

Figure S44. Bi-23-acetyldiosgenin ester (**10a**) in EtOAc showing strand-shaped structures.

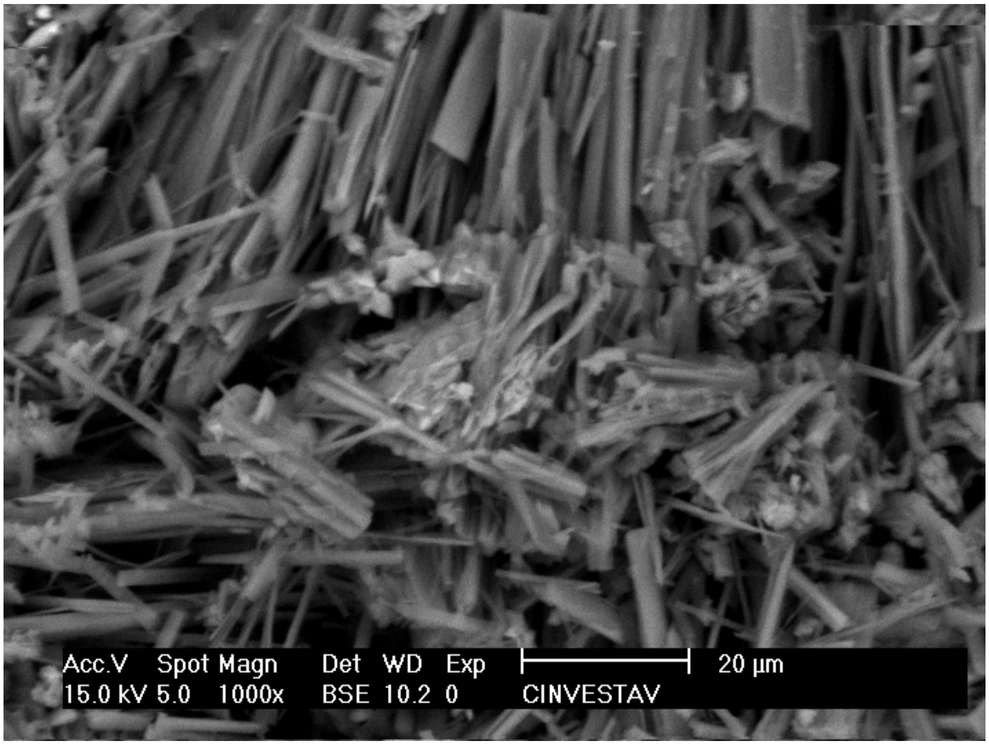

Figure S45. Bi-23-acetylhecogenin ester (**10b**) in EtOAc showing strand-shaped structures.

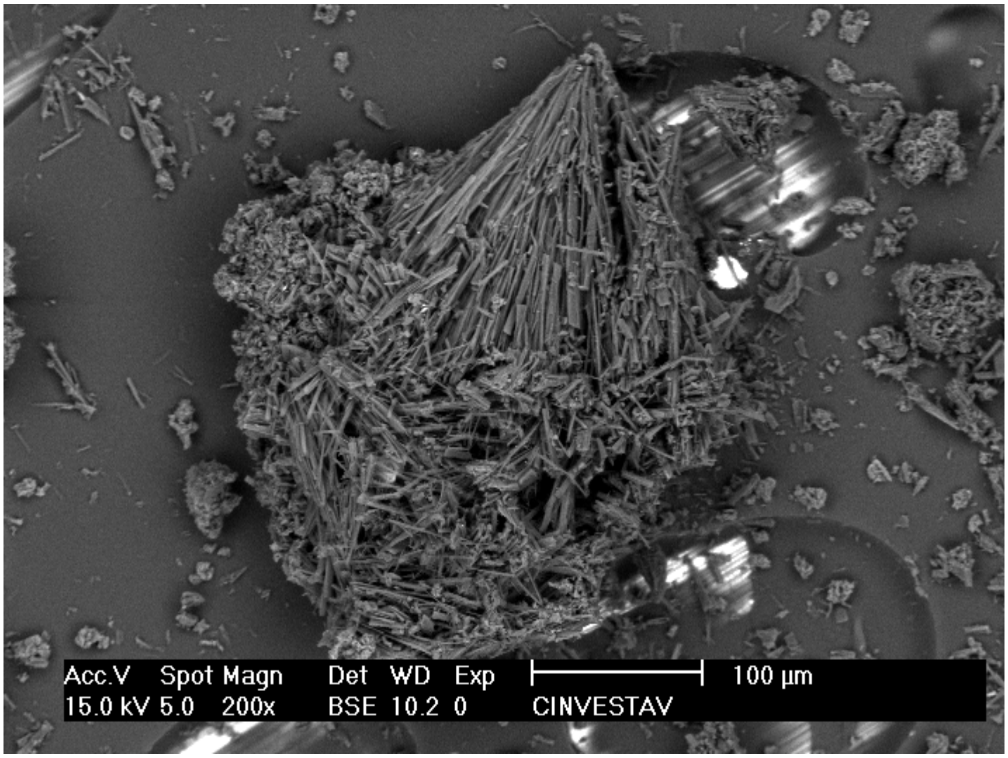

Figure S46. Bi-23-acetylhecogenin ester (**10b**) in EtOAc showing strand-shaped structures.

Powder X-ray diffraction (PXRD) analysis.

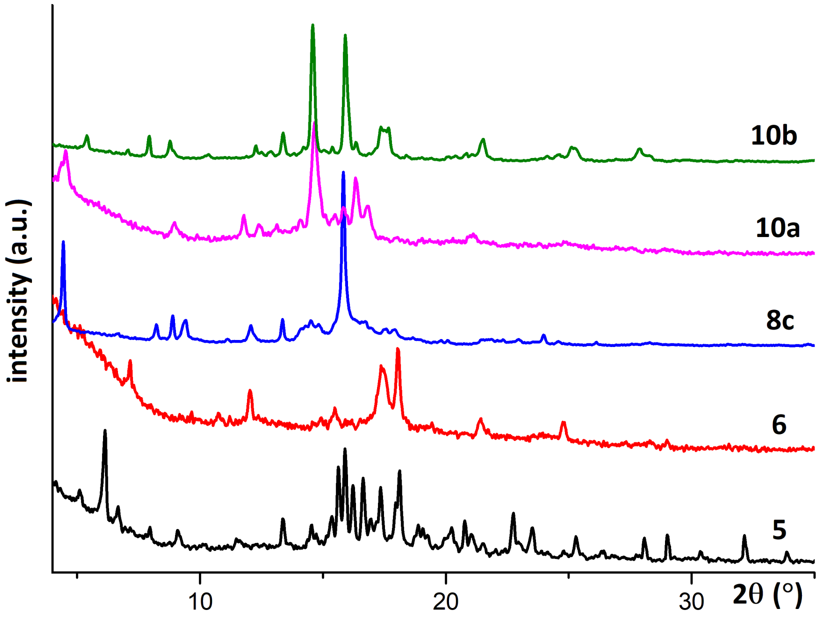

Figure S47. Powder X-ray diffraction patterns for raw materials of steroidal dimers **5**, **6**, **8c**, **10a** and **10b**. Patterns were collected with the Cu-*K*α radiation, and are uncorrected for amorphous contribution.

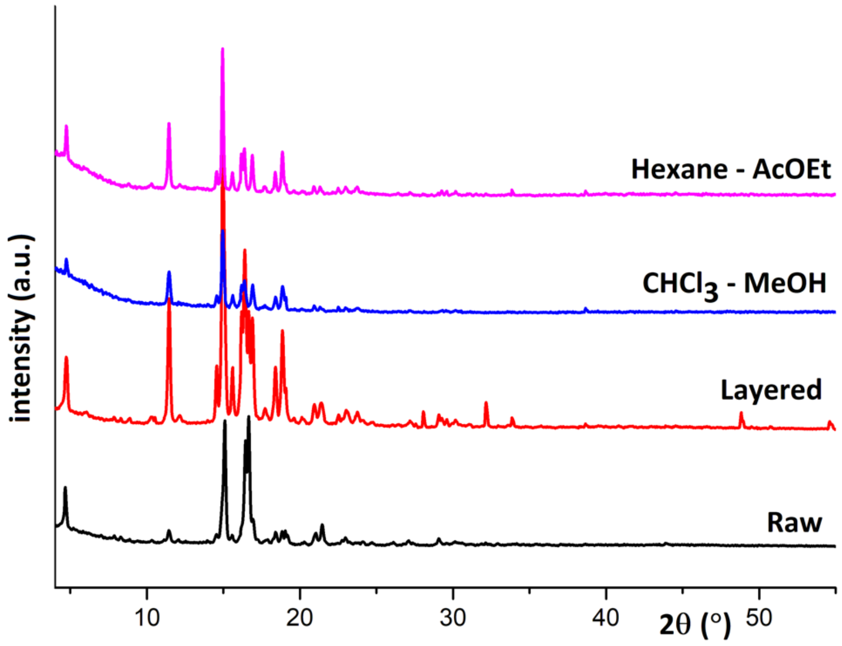

Figure S48. Comparison of the X-ray diffraction patterns of bidiosgenin ester (**8a**) under different conditions: raw material, layered material, dimer in contact with chloroform-methanol, and with hexane-ethyl acetate. Patterns were collected with the Cu-*K*α radiation, and are uncorrected for amorphous contribution. Note the intensity variation for the peak at 2θ = 11.5°, as a consequence of the self-organization of the steroidal dimer.
